# Enhancing spatial inference of air pollution using machine learning techniques with low-cost monitors in data-limited scenarios†

**DOI:** 10.1039/d3ea00126a

**Published:** 2024-01-25

**Authors:** Leonardo Y. Kamigauti, Gabriel M. P. Perez, Thomas C. M. Martin, Maria de Fatima Andrade, Prashant Kumar

**Affiliations:** a Departamento de Ciências Atmosféricas, Universidade de São Paulo Brazil leonardo.kamigauti@usp.br; b Global Centre for Clean Air Research (GCARE), School of Sustainability, Civil and Environmental Engineering, Faculty of Engineering & Physical Sciences, University of Surrey Guildford GU2 7XH Surrey UK; c Department of Meteorology, University of Reading UK; d MeteoIA São Paulo Brazil; e Institute for Sustainability, University of Surrey Guildford GU2 7XH Surrey UK

## Abstract

Ensuring environmental justice necessitates equitable access to air quality data, particularly for vulnerable communities. However, traditional air quality data from reference monitors can be costly and challenging to interpret without in-depth knowledge of local meteorology. Low-cost monitors present an opportunity to enhance data availability in developing countries and enable the establishment of local monitoring networks. While machine learning models have shown promise in atmospheric dispersion modelling, many existing approaches rely on complementary data sources that are inaccessible in low-income areas, such as smartphone tracking and real-time traffic monitoring. This study addresses these limitations by introducing deep learning-based models for particulate matter dispersion at the neighbourhood scale. The models utilize data from low-cost monitors and widely available free datasets, delivering root mean square errors (RMSE) below 2.9 μg m^−3^ for PM_1_, PM_2.5_, and PM_10_. The sensitivity analysis shows that the most important inputs to the models were the nearby monitors' PM concentrations, boundary layer dissipation and height, and precipitation variables. The models presented different sensitivities to each road type, and an RMSE below the regional differences, evidencing the learning of the spatial dependencies. This breakthrough paves the way for applications in various vulnerable localities, significantly improving air pollution data accessibility and contributing to environmental justice. Moreover, this work sets the stage for future research endeavours in refining the models and expanding data accessibility using alternative sources.

## Abstract 2

Our novel approach leverages accessible datasets and deep learning to achieve accurate air quality modeling in resource-limited environments.

## Graphical abstract

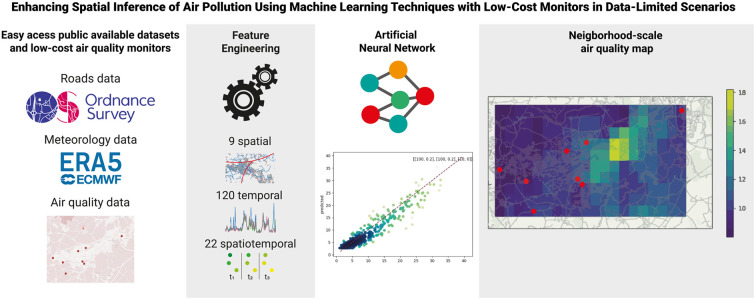

**Environmental significance**

In the field of air quality and machine learning, most research focuses on places with abundant data, often sidelining regions with limited resources like low-income countries and cities. This happens because better results are often achieved when using local-specific datasets. Our study aims to balance this by creating detailed maps of particle distribution in Woking, UK. We used deep learning and easily available datasets like ERA5's global reanalysis and local road data from Ordnance Survey, along with affordable Plantower PM sensors. Despite some limitations in how well these datasets match the location or how reliable they are, our model performed impressively, with an RMSE of less than 2.9 μg m^−3^. Our paper explains different strategies we used to handle data gaps, showing that powerful machine learning can work even when resources are limited.

## 1. Introduction

Environmental justice necessitates the collection and dissemination of environmental data in all communities. Longdon^1^ discussed the importance of Environment Data Justice (EDJ) as the information becomes more and more common in our everyday lives, and unfair treatment or bias caused by technology happens more often. However, the high cost associated with reference air pollution monitors poses a fundamental barrier to low-income locations. This issue is particularly concerning given the significant global impact of air pollution, with approximately 4.2 million annual deaths attributed to its effects.^2^ Moreover, populations in developing countries bear the greatest burden of exposure to air pollution. Hajat *et al.*^3^ in a review described studies conducted in North America which have consistently shown that areas where low-socioeconomic-status communities live tend to have higher concentrations of criteria air pollutants. To address this challenge, the establishment of large networks of low-cost monitors (LCM) has emerged as a potential solution to enhance data collection in low-income countries.^4^

In terms of data availability, machine learning (ML) solutions have been developed in recent years to spatially predict air pollution dispersion and other atmospheric properties. These ML models leverage reference pollutant monitoring networks along with supplementary datasets specific to each city, thereby reducing the reliance on a high number of monitors in a given location. Hu *et al.*^5^ compared a wide range of ML models for carbon monoxide spatial inference. The features used in their models included carbon monoxide concentration, geographic coordinates, hour of the day, day of the week, and season. Support Vector Regression exhibited the best overall performance in their evaluation. Similarly, Song *et al.*^6^ emphasized the importance of advanced feature engineering and utilized gradient boost decision trees with real-time traffic conditions and social media usage data. More recently, Martin *et al.*^7^ employed modern Neural Network models to downscale meteorological variables, demonstrating the applicability of their approach to air pollution variables. Their method involved principal component analysis (PCA) on atmospheric variable observations, enabling spatial and temporal predictions through the separation of loadings and scores. The best-performing ML model in their study was an artificial neural network (ANN) configured with two fully connected hidden layers, employing rectifier linear units (ReLU) and a dropout layer for regularization.

By modelling the relationship between reference monitors and complementary datasets containing information about local pollution sources, meteorological conditions, and background pollution, ML approaches improve the spatial resolution and accuracy of air pollutant concentration estimates. Martin *et al.*^7^ showed that Artificial Neural Networks (ANN) and Extreme Quantile Mapping (EQM) techniques significantly improve predicting the occurrence of extreme events. These methods have been particularly effective in capturing the variability associated with events like the formation of intense cold air pooling or heavy precipitation in valleys. However, existing ML air pollution models heavily rely on extensive city-specific data, such as smartphone data and real-time traffic information, which are often unavailable in many low-income locations. This limitation is evident in prominent ML models developed thus far.

In this article, we showcase the application of recent ML techniques using particulate matter (PM) measurements obtained from an LCM network managed by a local community in Woking, United Kingdom. We trained an ANN model to learn the relationships between the nearby PM concentrations, meteorology and nearby roads using the LCM network, the ECMWF's ERA5 reanalysis dataset^8^ for meteorological variables and the UK government's local roads data. It allowed the calculation of the PM concentrations for 200 points on Woking. Unlike previous air pollution studies employing ML, our proposed approach solely relies on widely available datasets, thereby facilitating the replication of our methodology in other locations and advancing the cause of data and environmental justice. While our study site is situated in a prosperous country, we selected Woking due to its status as a small town with identifiable emission sources, which offers an ideal setting for evaluating ML models. Importantly, our approach is not limited to LCM networks but can be readily applied to other networks comprising reference monitors.

## 2. Methods

**Fig. 1:**
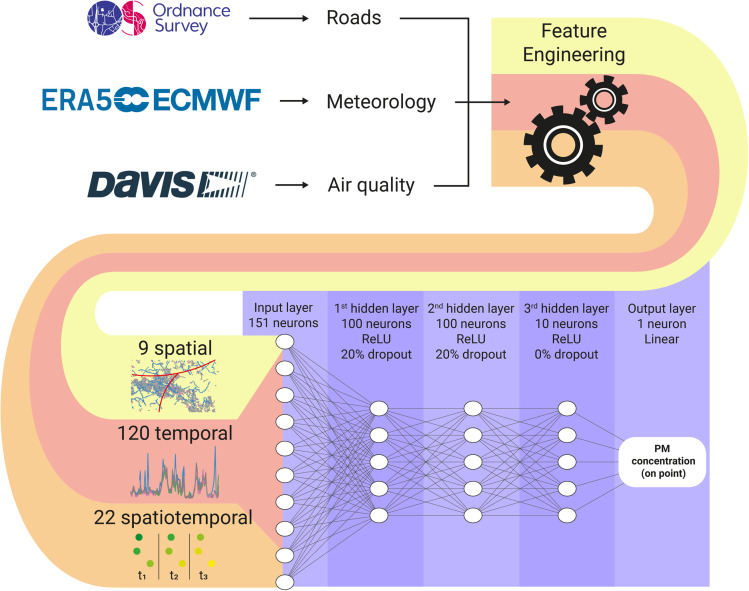
Flowchart of the methods. To calculate the particulate matter on the point, the data collected from the sources is used in the feature engineering process that produces 151 features per point. The features are inputted in the neural network composed of 3 hidden layers using ReLU activation functions. The first and second hidden layers has 100 neurons each and 20% dropout rate. The last hidden layer is a bottleneck with 10 neurons and 0% dropout.

The study utilized ambient PM concentration data from a citizen-led network of Low-Cost Monitors (LCM) located in Woking, a typical town in the UK. The LCM data was collected through a joint initiative between Woking's local citizen network, the Woking Green Party, and the Guildford Living Lab. Meteorological data from the fifth generation of the European ReAnalysis (ERA5) dataset and road data from the Ordnance Survey were also incorporated. To calibrate the LCM data, a ridge polynomial regression was employed, with temperature and humidity as support variables. The PM concentrations over Woking were modelled using a fully connected neural network (Fig. 1). The details of the methodology are described in the following sections, and the computer code used in this work is available on GitHub. Additional technical information can be found in the ESI.†

### 2.1 Monitors' description

Monitors in the citizen-led network consisted of 8 Davis® AirLink monitors equipped with Plantower PMS7003 sensors^9^ nominally capable of measuring PM concentrations in modes of 1, 2.5, and 10 μm (PM_1_, PM_2.5_, and PM_10_, respectively). The sensor's working principle is based in laser scattering, where the particles in a measuring cavity are radiated by a laser, and a light detector is positioned in the cavity, forming an angle with the laser, detecting the light scattered by the particles. The electric signal of the light sensor is then amplified and converted to particle concentrations using Mie theory internal calculations in the sensor's microprocessor.^9^ The sensor and monitor documentation do not explicit the method used to separate the PM fractions, and there is other LCM that simply define a ratio between the PM sizes (*e.g.* The sensor DSM501A, Samyoung S&C Co., Ltd), thus not being a direct source of PM concentrations in different sizes by itself. It would be mitigated using a colocation using ambient air in the local, following international standards,^10,11^ however, we did not had resources to do field calibrations. Instead, we performed a chamber calibration of the monitors using not only the PM concentrations reported by the models, but also temperature and relative humidity as input parameters. The monitors' performance was characterized by a comparison between the sensors and a reference sensor (Grimm EDM 107 optical particle counter, Grimm-Aerosol GmbH & Co., Germany) inside the ENVILUTION® Chamber according to the protocol described by Omidvarborna *et al.*,^12^ with details in the ESI.† The monitors provided data through the WeatherLink platform and had a Pearson's correlation of at least 0.79 with reference monitors, with an RMSE of 7.4 μm m^−3^. The monitors also recorded relative humidity with an accuracy of ±2% and temperature with an accuracy of ±0.3 °C. This study evaluated the model's performance and calibration, and the results are presented in the ESI.†

### 2.2 Monitors' location

**Fig. 2:**
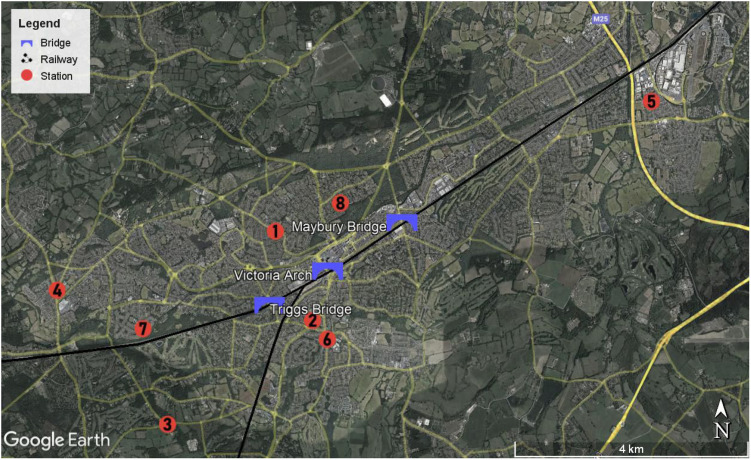
Monitor locations in Woking, UK. The locations are marked by the numbered red mark. The main bridges are indicated in blue. The M25 road is in the northeast and the A3 road is in the southeast. The M3 road is not on the map. Basemap from Google.

The monitors were strategically distributed throughout Woking, located in Surrey County, United Kingdom (Fig. 2). Woking is situated at the southwestern edge of the Greater London Urban Area and experiences an average temperature of 13.45 °C (with a standard deviation of 4.77 °C) and an average relative humidity of 82.28%. The city has relatively slow wind speeds, averaging 3.56 m s^−1^ (with a standard deviation of 1.58 m s^−1^). Woking is part of the London Commuting Belt and is intersected by major roads such as the London Orbital Motorway (M25), A3, and M3. The city's topography is relatively flat, and it is divided by a railway into north and south, connected by three main bridges: Victoria Arch, Maybury Hill and Monument Road, and Triggs Lane. During the study period, the Victoria Arch area was affected by the Victoria Arch Widening Scheme, which led to a redirection of traffic to other bridges in the city.

### 2.3 Auxiliary datasets

#### 2.3.1 Meteorological fields

Meteorological data was obtained from the European Centre for Medium-Range Weather Forecasts (ECMWF) ERA5 reanalysis dataset.^8^ The dataset, with a spatial resolution of approximately 31 km and a temporal frequency of 1 hour, was converted to daily frequency for the study. The data, available with a 5 days lag, could be downloaded from the ECMWF's CDS system (available at https://cds.climate.copernicus.eu/) in netCDF format.

#### 2.3.2 Road maps

Road data was sourced from the Ordnance Survey (OS) Open Roads dataset, which is a free dataset provided by the UK government. The dataset, obtained in GeoPackage format, includes georeferenced vectors categorized by road type. To calculate a road density index, the road data was rasterized with a resolution of 50 × 50 m, and the vector points were summed within each grid cell. The road density around each pollution monitor or map point was determined by summing the raster points in a 200 m^2^ square.

### 2.4 Model

A Multi-Layer Perceptron (MLP) was chosen to capture the spatial nuances of the data and relate temporally constant variables, such as road density, with the temporal variables. The MLP architecture consisted of 3 hidden layers, with the first two layers comprising 100 neurons each and a dropout rate of 20%,^13^ while the last layer consisted of 10 neurons without dropout. ReLU activation functions were used in the hidden layers, and linear activation functions were used in the output layer (Fig. 1). The network was trained using Adam optimization,^14^ and a batch size of 24 samples and 20 epochs. The same set of hyperparameters was used for each PM size (PM_1_, PM_2.5_, and PM_10_). Five of the eight monitors' data were used for training, and the remaining three monitors' data were used for model evaluation. The monitors chosen for evaluation were the 2, 7 and 8, as their PM concentrations are close to the average concentrations of the city, the monitors are surrounded by others, and the PM concentrations distributions does not indicate important local sources (see ESI†). For the training, the last 20% of the data (in time) was separated to validation, in which the model's loss (mean absolute error, MAE), and monitoring metrics (MAE and mean squared error; MSE) was calculated at the end of each epoch. Being *y* the target value and *ŷ* the calculated value, MAE and MSE are calculates as follows:1
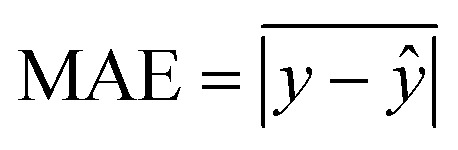
2
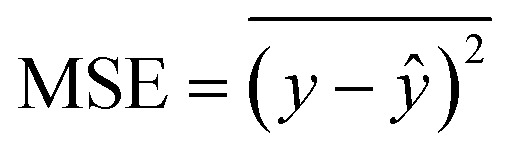


The feature engineering process was applied in the training and test datasets separately, making sure that there were no information leaks between them, *i.e.*, the evaluation dataset does not have information about the other monitors in their features, and *vice versa*. In total, 151 features were used as the input of the model from different sources related to spatial and temporal information (Table S2†). Regarding the final PM distribution map over the study area, the map features were calculated for each point of a 10 × 20 grid of 600 m resolution.

The temporal features were obtained with one-hour frequency and then resampled to one-day frequency using 24 hours average. The temporal-only features were: (i) day of the week, (ii) month, (iii) ERA5 variables, (v) and the average PM concentration of the monitors. The nearest data point of ERA5 to the city centre was chosen to represent the local meteorology. The 8 data points around the city in the cardinal and ordinal directions were inputted in the model as well.

The spatiotemporal features were composed of the 3 nearest monitoring monitors' PM concentrations (in all PM sizes; excluding the monitor itself in the training dataset), distances, angles between the point and the monitors, and a parameter of concordance between the wind direction and the monitor angle (0 if the monitor is downwind and 1 if is upwind); the IDW interpolation of the 3 nearest monitors (weight = 2, and with the same considerations as before); and the difference between the average PM concentration and the IDW. The PM concentrations were used as the target variable to train and evaluate the models.

The spatial-only features were the density of roads in a 200 m^2^ square around each monitor subdivided by road type. The road data from OS has 8 categories based on road usage: A Road, B Road, Local Access Road, Local Road, Minor Road, Motorway, Restricted Local Access Road, and Secondary Access Road. The data were available as vector files covering the areas SU95, SU95, TQ05, and TQ06 of the Ordnance Survey National Grid reference system. Each category vector was rasterized to 4 m resolution grids containing the counting of each road in the area. Then, the pixels in a 200 m^2^ square around each monitor were summed. This number is proportional to the local road density of each category.

Model evaluation employed metrics such as RMSE, Symmetric MAPE (SMAPE), coefficient of determination (*R*^2^), and Pearson's *r*. A visual comparison of the model's PM distribution with the monitor's data was performed as a sanity test. The sensitivity of the model to each input feature was estimated using a One-at-a-time (OAT) sensitivity analysis adapted from Loucks *et al.*^15^ which provides insights into feature importance and helps guide future model design. More details on the OAT analysis can be found in the ESI.† Being *y* the target value and *ŷ* the calculated value, and *n* the number of samples, the model evaluation metrics are calculated as follows:3
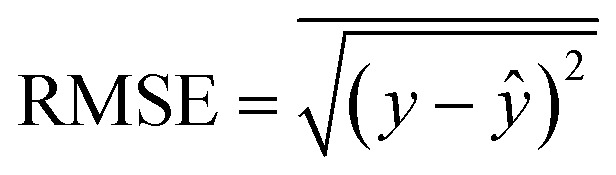
4
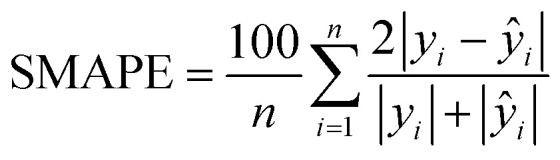
5
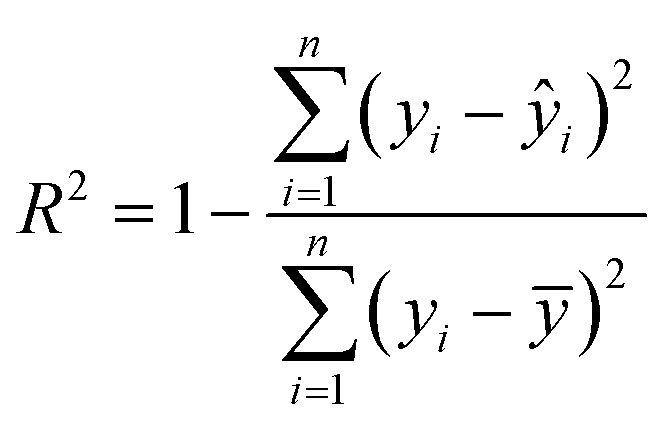
6
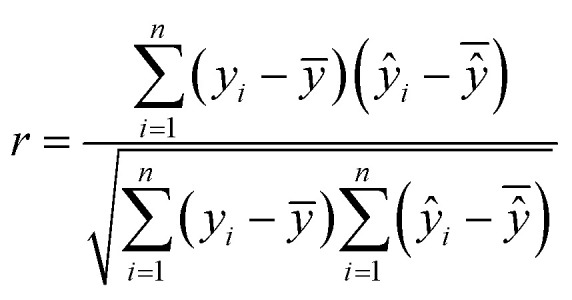


### 2.5 PM data description

The PM data (PM_1_, PM25, PM_10_) cover a period of 6 months, from July to December 2021 (Fig. S11†). A short statistical description of the PM size-segregated data is in Table S4.† The data is not available for all stations in July, specially from Monitor 7 due to installation procedure performed in that month. To describe the spatial variations of the monitors, an index of local pollution was calculated by subtracting the average concentration (details in the ESI†). Fig. S12† shows a heatmap of the average difference between each monitor for each interquartile, where the rows and columns are respective to the monitor's number. The diagonal of the heatmap is zero because the difference between the monitor and itself is zero. The spatial variations are to be subtle across the monitors, with averages being between −2.5 and 2.5 μg m^−3^. Monitors 6, 2, and 1 are above the average, monitors 8 and 5 are very close to the average and monitors 7, 4, and 3 are below. The spatial differences increased with the intensity of the PM concentrations (Fig. S12†), reaching over 6, 8, and 8 μg m^−3^ for PM_1_, PM_2.5_ and PM_10_ respectively, indicating the dominance of local sources in those events.

## 3. Results and discussion

### 3.1 Models' evaluation

**Fig. 3:**
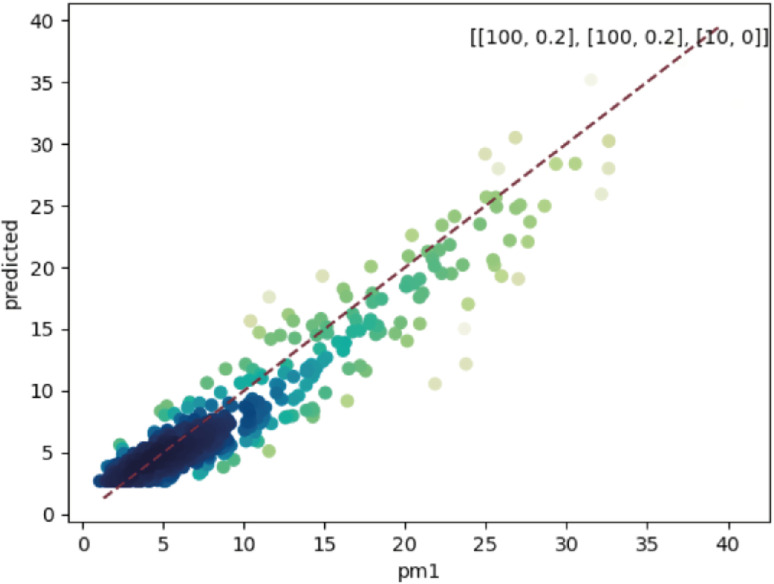
Comparison between the values of PM_1_ predicted by the model (*y*-axis) and the actual values (*x*-axis) in the evaluation dataset. The colour of the dots is proportional to the density of points. The dashed line is the 1 : 1 line. The number of neurons per layer and dropout rate are in the top-right corner in the format “[number of neurons, dropout rate]”.

To evaluate the models, we compared the PM concentrations data from the evaluation monitors with the models' outputs with inputs calculated on the location of the evaluation models, during the same period and agnostic of the evaluation monitors data. Fig. 3 shows the results of the models' evaluation (figures referring to PM_2.5_ and PM_10_ are in the ESI† because they are very similar to PM_1_ figures). The metrics are presented in Table 1. The models performed similarly regardless of the PM size. As stated in the Section 2.1, it may be due the method of PM size differentiation of the monitors, but also may be attributed to the predominance of PM_1_ in the region, with coarser PM sizes representing 10% or less of the total PM mass (see Table S4†).

**Table 1:** Evaluation metrics of the models

Model	RMSE [μg m^−3^]	SMAPE [%]	*R* ^2^ [adim.]	*r* [adim.]
PM_1_	2.57	25.31	0.88	0.95
PM_2.5_	2.89	26.82	0.90	0.95
PM_10_	2.36	22.23	0.93	0.97

**Fig. 4:**
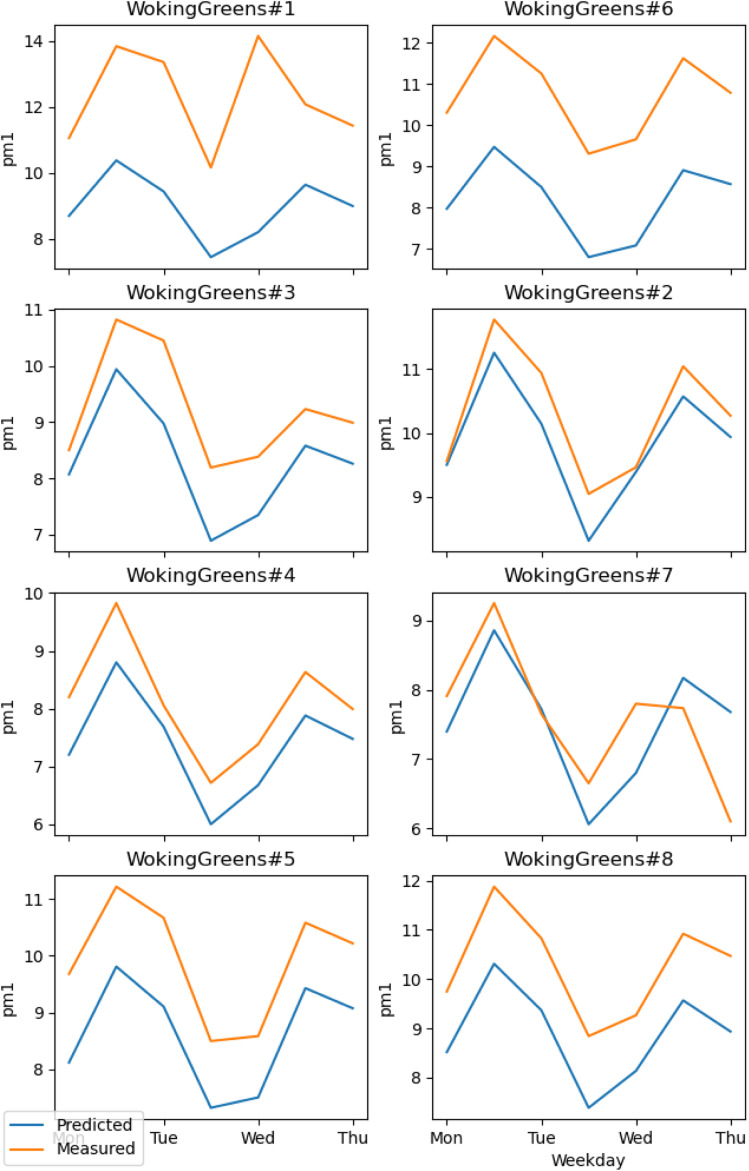
Comparison of the weekly variation of PM_1_ between the model and the dataset. Stations numbers 2, 7 and 8 are the evaluation ones. The other belongs to the training set.

The RMSE shows an error within the spatial differences in the dataset, indicating a capacity to distinguish the spatial information, especially in highly polluted scenarios, what is relevant in terms of air pollution alerts to population. The SMAPE indicates a bias in the model. This bias indicates an underestimation of the concentrations. The *R*^2^ shows that most of the variance of the data is explained by the model. It indicates that the model can describe major processes that dominate the variance. Pearson's *r* indicates a strong linear correlation between our model and the evaluation data, suggesting a linear fit. In comparison to the model developed by Song *et al.*,^16^ which achieved RMSE of 13.17 μg m^−3^, SMAPE of 14.65%, and *R*^2^ of 0.91 for PM_2.5_, the models developed in our study performed better. The RMSE was more than 10 μg m^−3^ lower, and the *R*^2^ was similar (except for PM_1_). However, the SMAPE of our models was on average 10.14 percentage points higher compared to Song *et al.*'s model. It is important to note that the average PM_2.5_ concentration in their study is around 40 μg m^−3^, as estimated by Song *et al.* Our concentration is almost four times lower than the levels observed in their study. The sanity test shows the ability of the model to replicate the overall shape of the weekly variation curve for all monitors and PM sizes (Fig. 4). There is no clear difference between the evaluation and training data in this test. In agreement with the SMAPE, the model consistently underestimates the concentrations, with no apparent effect on the day of the week or PM concentration. The differences between the model and the measurements are within the RMSE in all cases.

The OAT analysis is not sensitive to non-linear relationships between the model features. Therefore, physical interpretations of the models' intern reasoning need to be taken cautiously. However, interpretations are valuable to future feature engineering in related applications. The sensitivity analysis (Fig. S17–S19†) indicates that all models used the monitors dataset as the primary source of the final prediction. It is expected because the monitors data are the most direct information of the PM concentration in the local. The meteorological variables of most influence were the boundary layer dissipation (BLD), boundary layer height (BLH), total precipitation, and mean total precipitation rate. The BLD is the amount of kinetic energy converted to heat due turbulence, inside the boundary layer. This turbulence is related to the mixing rate of the PM, which influences how much a local event spreads and dilutes not considering the transport by wind. The BLD also influences the pollutants exchange between the stable boundary layer and the residual layer formed at night, which can trap the nocturnal emissions closer to the ground. It is especially problematic as the local community burn wood in fireplaces for heating in the winter. The BLH dictates the volume of atmosphere available for easy dispersion of pollutants, directly influencing the concentration on the surface. Precipitation governs the process of wash-out, being directly related to PM removal, especially in larger particle sizes.

Regarding the differences between the models in the OAT analysis, for the PM_1_ model, the northeast meridional wind speed was especially influential, being comparable to the interpolation of PM_2.5_ and the average PM_1_. It shows a high sensitivity of PM_1_ to windspeed, indicating a high importance of the transport of PM_1_ from far sources. The northeast direction is the closes to London, which is potentially the main far source of the region. The PM_2.5_ model was especially sensitive to northwest total precipitation, with influence near the second nearest PM_10_ concentration and the minor roads. The total precipitation is expected to be influential in the model, however, it is not clear why the northwest region was the one with most impact in the model. The PM_10_ model also presented a meteorological variable among the PM concentrations, with boundary layer dissipation at the northeast and southwest between the interpolation of PM_2.5_ and the nearest PM_10_ concentration. The models were less sensitive to the road data than the monitors' data, and there were more differences between the road types than the monitors' features. It is expected, as the monitor data are directly related to PM concentrations, and roads are indirect. The higher difference in road types indicates that the model learned different relations the roads can have with the monitors' data. The PM_1_ and PM_2.5_ models were more sensitive to minor roads, and B roads. It can be attributed to more variable accelerations in the smaller roads, and to the residential zones of the city. The PM_10_ model was sensitive to motorways, B roads, and A roads. It can be attributed to soil resuspension by high velocity heavy vehicles in higher speed roads.

### 3.2 Pollution map

**Fig. 5:**
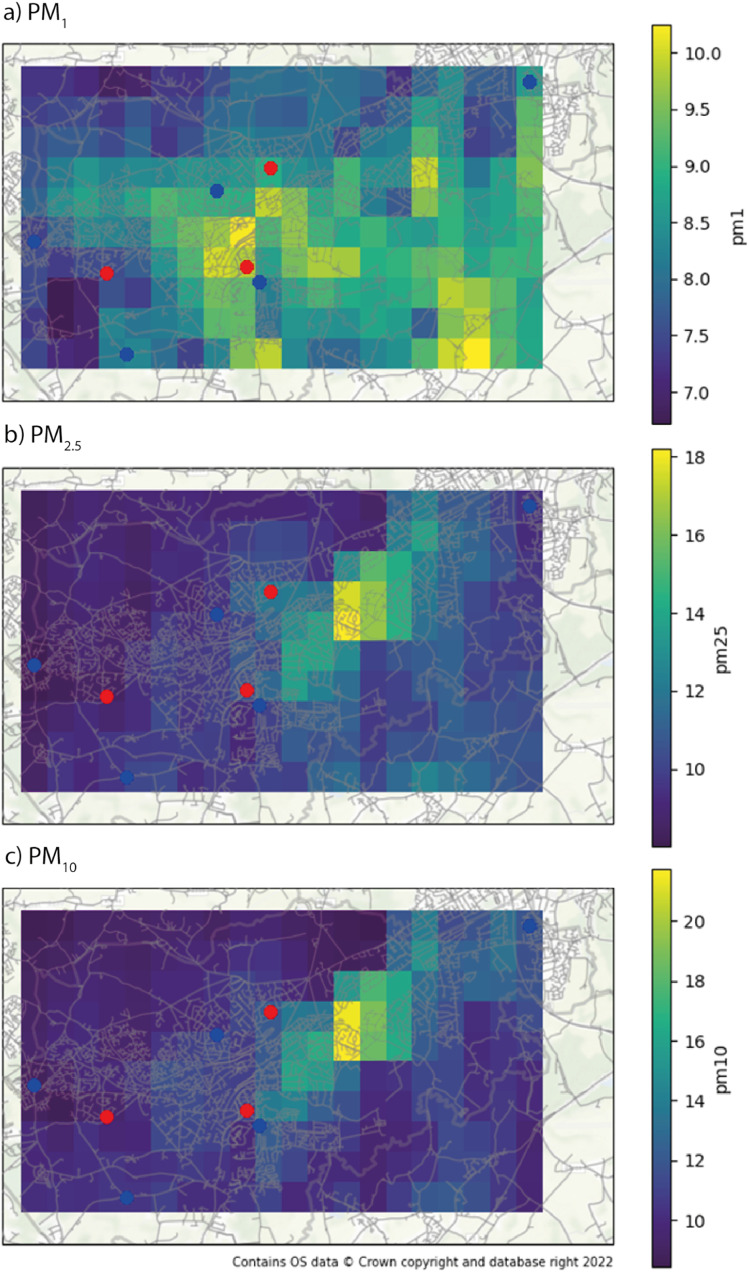
Average PM_1_ (a), PM_2.5_ (b), and PM_10_ (c) over Woking, UK in the timeframe of the study. The training monitors' locations are represented by blue dots and the evaluation monitors are in red.

The main product of the model is the 24 h PM average over Woking (Fig. 5). The map was produced in 10 × 20 squares of 600 × 600 m each. We used the models to calculate each point in the map, for each day in the dataset, and then, we calculated the average PM concentration for each point in the map. The calculation of the features for each map point for all days in the dataset was slow (more than 3 days of computation), hindering the plot of a more detailed map. The maps used all data available in the study, presenting the average PM concentrations in a period of 6 months, from July to December 2021. The maps show different distributions of PM, being PM_1_ higher in the city centre, near the A3 highway (that connects London to Woking, in the southwest), at the south exit of the residential area of Pyrford (at the west of Woking centre), and at the B380 and Guildford Road in the south exit of the residential area of Westfield (at the south of Woking centre). The Guildford Road area is already identified as an Air Quality Management Area (AQMA) by the Woking Borough.^17^ The AQMAs are areas not expected to meet the government's limits for air quality. The distribution of PM_1_ is more spread than the other sizes, with less variance. It indicates the presence of an important background source such as secondary particle formation, which is expected due to the strong presence of vegetation inside and around Woking and is in line with other source apportionment studies in the area.^18^ The model does not have dynamic information about the precursors, which could improve its fine fraction resolving and lower the *R*^2^. The distribution however is close to the nitrogen dioxide model requested by the local authorities in 2019 (ref. 18–20) to the Cambridge Environmental Research Consultants (CERC). It indicates an interaction between the vehicular emissions and the background precursors in the secondary formation.

PM_2.5_ and PM_10_ have similar spatial distribution due to their similar small concentrations (there is a low mass of particles between 2.5 and 10 μm). Its peak concentration is in East Hill/College Road, which leads to Woking's shopping centre, and a bridge that crosses the train line that divides the city. It is important to note that the city centre bridge (Victoria Arch) was blocked due to the Victoria Arch Widening Scheme. The traffic was diverted to the bridge near the peak concentrations of PM_2.5_ and PM_10_ and to the bridge in Triggs Ln. in the west of the city centre. There are also local maximums near the A3 and M25 highways. These pollution maps showing hot spots of pollution near roads and traffic intersections are consistent with other authors.^21^ The concentration values are compatible with the PM model requested by the local authorities in 2019 to CERC,^18–20^ however, the dispersion is different, lacking a higher concentration around the Victoria Arch bridge. A probable explanation is that CERC used a model based on Surrey's Department of Transport Traffic Model,^18^ which was ingested with data from Surrey Traffic Surveys. However, in the region of Victoria Arch, there is only four days of data collection in 2019 (13 and 15 May, 9 and 11 September^22^).

### 3.3 Early model versions

The initial tests of the ANN explored the lower and upper limits of the number of layers, neurons, and dropout factors. The established limits were from 2 to 4 layers, from 10 to 200 neurons, and from 0 to 0.25 dropout factor. The models showed better performance when the dropout factor was set to 0 in the final layers.

Previous configurations generated many models with worse metrics performances. The main cause of failure was the use of a one-hour timestep. Figures of the evaluation of the best model trained with one-hour timestep are presented in the ESI.† The low *R*^2^ (of 0.54) and high SMAPE (53.56) indicate that the model could not account for major sources of variability in the physical system. The sanity tests revealed that the model could not reproduce the amplitude of the variance in the hourly variation over the hours, severely underestimating the concentration of the pollutant in the night period. It may be caused by the imprecision in the ERA5 planetary boundary layer height data, which is not derived from direct measurements. The use of a timestep of one day reduced the complexity of the model while at the same time maintaining its usability as most proposed air pollution limits use daily intervals.

## 4. Conclusions

The findings of this study highlight the potential of machine learning techniques to generate valuable spatial information from low-cost pollution monitors, even in scenarios with limited data availability. By employing feature engineering and an appropriate timestep, we were able to develop effective neighbourhood-scale pollution dispersion maps within a reduced budget. These machine learning models have the potential to facilitate pollution monitoring applications in economically vulnerable areas, thereby contributing to environmental justice and enhancing air pollution data accessibility.

In the case of Woking, UK, our model successfully identified areas with high pollution levels associated with local traffic. However, it is important to note that achieving these results required multiple iterations and adjustments. We identified the influence of the planetary boundary layer as a significant challenge for the model, despite incorporating the layer height information from the ERA5 dataset. This indicates a weakness in the model's sensitivity to errors in the features dataset. To address this, future versions of the model will focus on improving data accessibility by utilizing the OpenStreetMap road database instead of the UK-focused OS dataset. Additionally, we plan to investigate the model's sensitivity and experiment with different preprocessing techniques prior to data input, aiming for further improvements and enhanced performance.

## Conflicts of interest

There are no conflicts to declare.

## Supplementary Material

EA-004-D3EA00126A-s001
