# Supplementary material for: Enhancing spatial inference of air pollution using machine learning techniques with low-cost monitors in data-limited scenarios
Source: Environ Sci Atmos. 2024 Jan 25;4(3):342–50. doi: 10.1039/d3ea00126a (PMC10938372; doi:10.1039/d3ea00126a)
Supplement: EA-004-D3EA00126A-s001 [file EA-004-D3EA00126A-s001.pdf]

Electronic Supplementary Material (ESI) for Environmental Science: Atmospheres.
This journal is © The Royal Society of Chemistry 2024

Enhancing Spatial Inference of Air Pollution Using Machine Learning Techniques with Low-Cost Monitors in Data-Limited Scenarios

Supplementary Information

Technical detailing of the softwares

Codes for dataset downloading and feature engineering are available in https://github.com/lokamigauti/surrey-networks. The study was made possible by utilising the Python 3.8 and the following libraries:

- Dataset download:
    - *cdsapi 0.5.1* (ECMWF)
- GeoPackage handling:
    - *geopandas 0.10.2* (Jordahl, et al., 2021)
    - *pandas 1.4.1* (Reback et al., 2022, McKinney, 2010)
    - *shapely 1.8.0* (Gillies et al., 2021)
- Vector rasterization:
    - *geocube 0.1.1* (Snow et al., 2022)
    - *rasterio 1.2.10* (Gillies et al., 2013)
    - *geopandas 0.10.2* (Jordahl, et al., 2021)
- Data manipulation and visualisation:
    - *numpy 1.21.4* (Harris et al., 2020)
    - *xarray 0.20.1* (Hoyer & Hamman, 2017, Hoyer et al., 2022)
    - *matplotlib 3.4.3* (Hunter, 2007)
- Machine learning:
    - *scikit-learn 1.0.1* (Pedregosa et al., 2011)
    - *keras 2.8.0* (Chollet et al., 2015)

Monitors' performance and calibration

The monitors' performance was characterised by a comparison between the sensors and a reference sensor (Grimm EDM 107 optical particle counter, Grimm-Aerosol GmbH & Co., Germany) inside the ENVILUTION® Chamber according to the protocol described by Omidvarborna et al. (2020). The monitors were placed in the chamber with the reference sensor for 2.5 hours. The internal conditions of temperature, relative humidity and PM concentrations were modified in the chamber (Figure S1). We calculated Pearson's correlation coefficient (r), coefficient of determination (R^2), root mean square error (RMSE) and mean absolute percentage error (MAPE). A summary of the average scores is in Table S1. The scores presented in detail are in Figures S2, S3, S4, and S5.

Table S1: Average scores of the uncalibrated monitors in relation to the reference sensor and standard deviation in parenthesis.

	PM_1	$PM_{2.5}$	PM_{10}
r	0.81(0.07)	0.75(0.09)	0.72(0.10)
R^2	0.44(0.20)	0.50(0.18)	0.53(0.17)
MAPE	0.46(0.16)	0.56(0.20)	0.70(0.25)
RMSE	17.40(6.18)	25.87(9.17)	25.52(9.03)

The PM measurements have a high correlation with the reference sensor, indicating a similar shape with the reference. Although, the R^2 and MAPE indicate considerable differences in the data and reference values. The RMSE quantified the differences as 17, 26 and 26 μg/m³ on average for PM_1, $PM_{2.5}$, and PM_{10} respectively.

A calibration was performed in order to improve the RMSE of the PM measurements (Figures S6, S7, and S8). We used the calibration data from the ENVILUTION® Chamber characterization. The PM measurement is reported in modes, containing redundant information about the particle sizes. For this reason, we calculated the concentrations in size ranges, rather than in size modes. The ranges used were 0-1, 1-2.5 and 2.5-10 μm (PM_{0-1}, $PM_{1-2.5}$, and $PM_{2.5-10}$, resp.) being the former equivalent to PM_1, the second derived from the subtraction of the $PM_{2.5}$ and PM_1, and the latter derived from the subtraction of the PM_{10} and the $PM_{2.5}$.
The equation was performed by applying a ridge polynomial regression using the following equation:

$$PM_{ref} = \sum_{var} wX_{P3},$$

Where X_{P3} is the scaled matrix of the polynomial combination without the constant term of the particulate matter, temperature and relative humidity until degree 3. The w is the Ridge Coefficients matrix, and the PM_{ref} is the particulate matter concentration measured by the reference sensor. The scaling was performed using the subtraction of the mean and division by standard deviation. The ridge coefficients were calculated using the following equation:

$$min_w = \left|\left|Xw - PM_{ref}\right|\right|_2^2 + \alpha\left|\left|w\right|\right|_2^2,$$

Where α is the complexity parameter. The second member of the equation controls the minimization, forcing it to maintain the regression coefficients as low as possible, which is reasonable to the physics underlying the problem. The complexity parameter prevents overfitting by restricting the size of the ridge coefficients. We selected α as 30 for $PM_{2.5-10}$, 10 for $PM_{1-2.5}$, and 5 for PM_{0-1} to maintain a good compromise between the representations of small variances, and at the same time improve the peak representation without overfitting the data. A weight vector was attributed to the measurement matrix in order to prioritise the data within the expected range of the local background. The weights were assigned as 1 to samples whose concentrations of PM_{10} were lower than 20 μg/m³ and 0.5 to the rest.

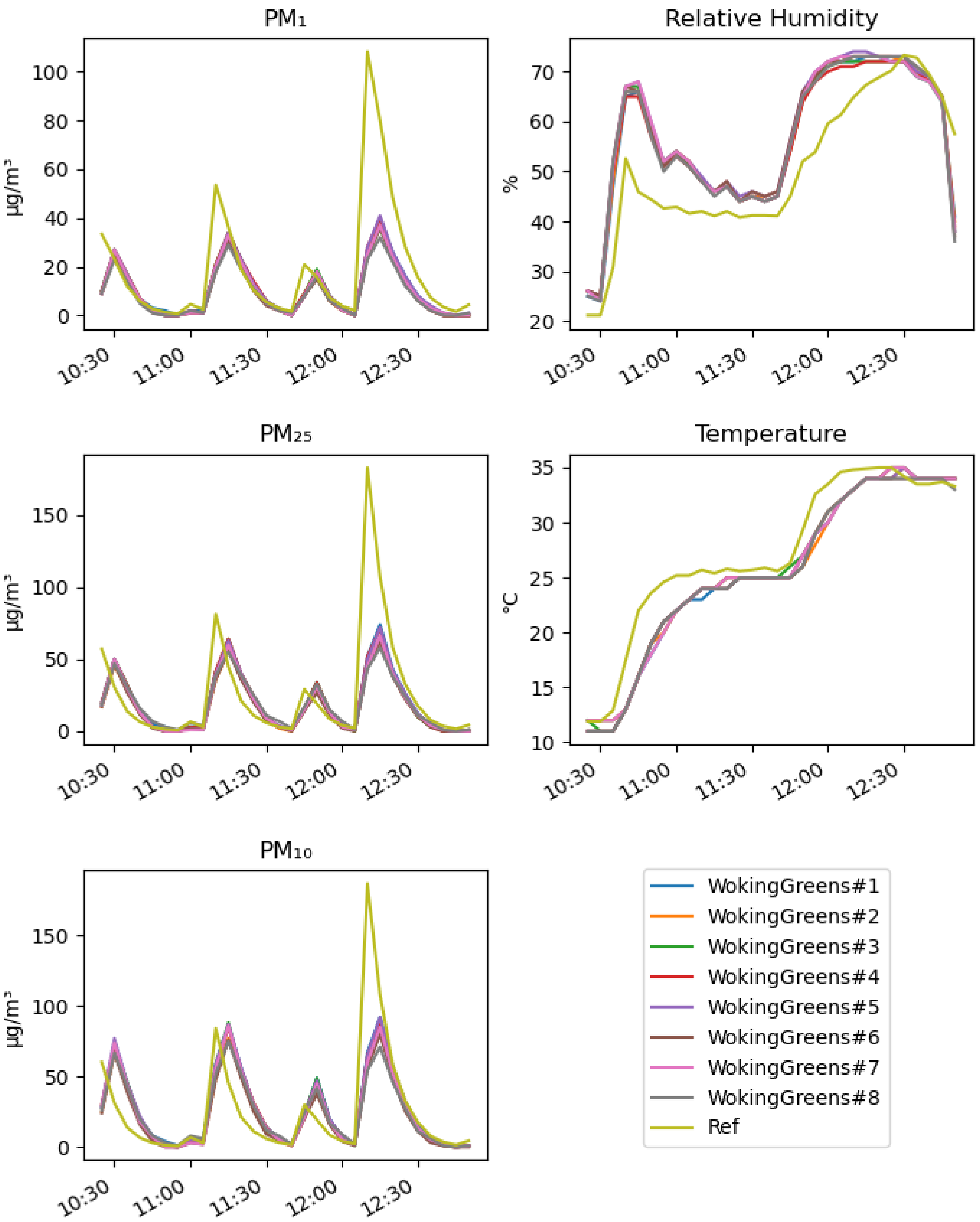

Figure S1: Chamber calibration data of the low-cost monitors. The variables inside the chamber (relative humidity, temperature, and particles) where individually and simultainiously controlled in order to make varied scenarios for calibration. The temperature and relative humidity where the chosen variables for calibration as they affect the sensors structure and internal geometry by thermal expansion and contraction, and the particles' size by condensation.

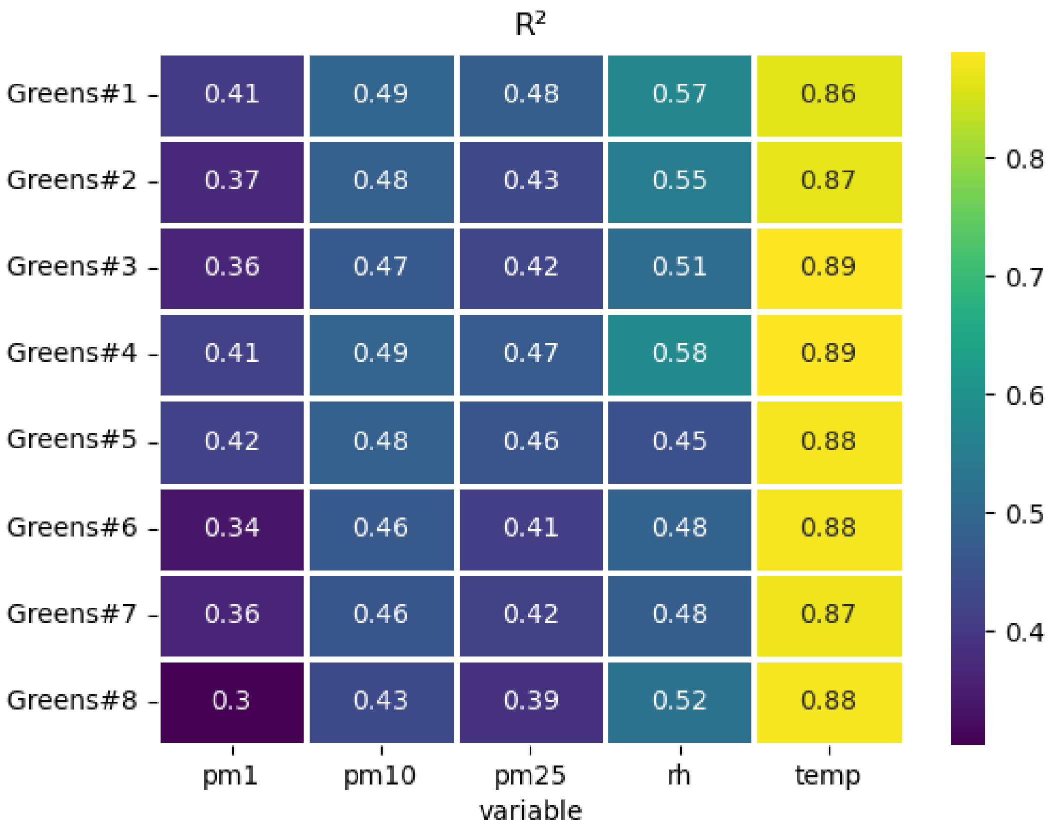

Figure S2: Heatmap of the coefficient of determination of the uncalibrated low-cost monitors by the measured variables.

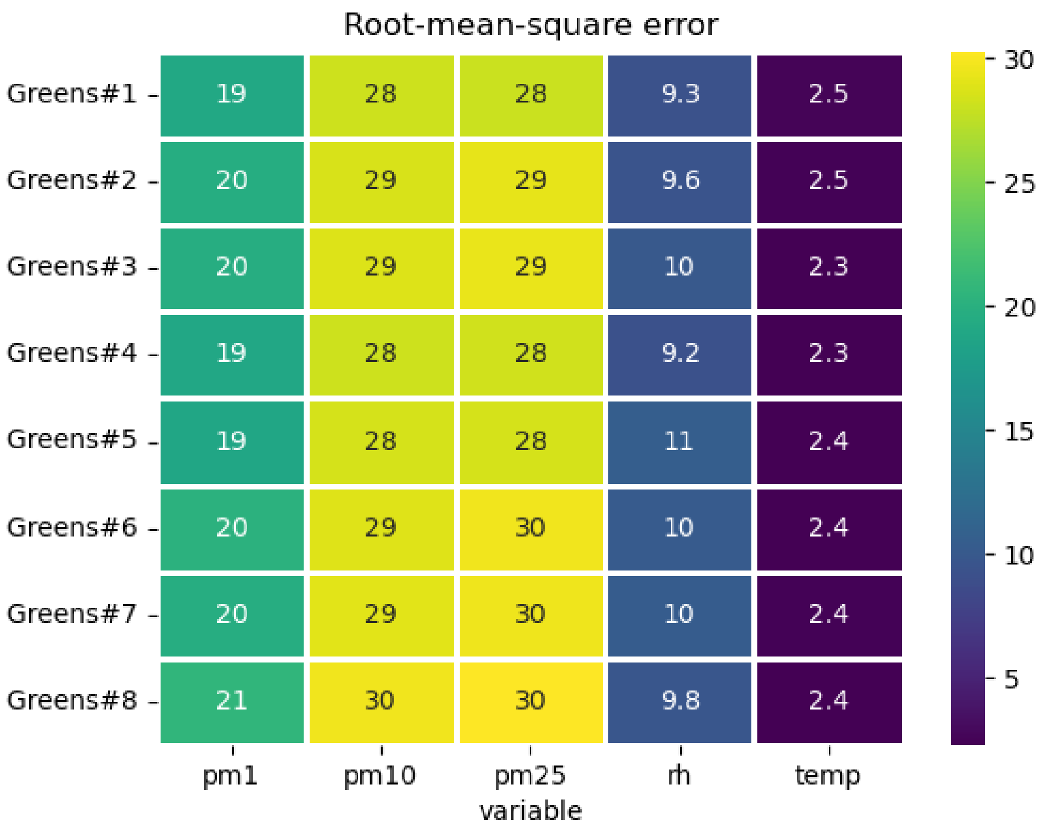

Figure S3: Heatmap of the root-mean-square error of the uncalibrated low-cost monitors by the measured variables.

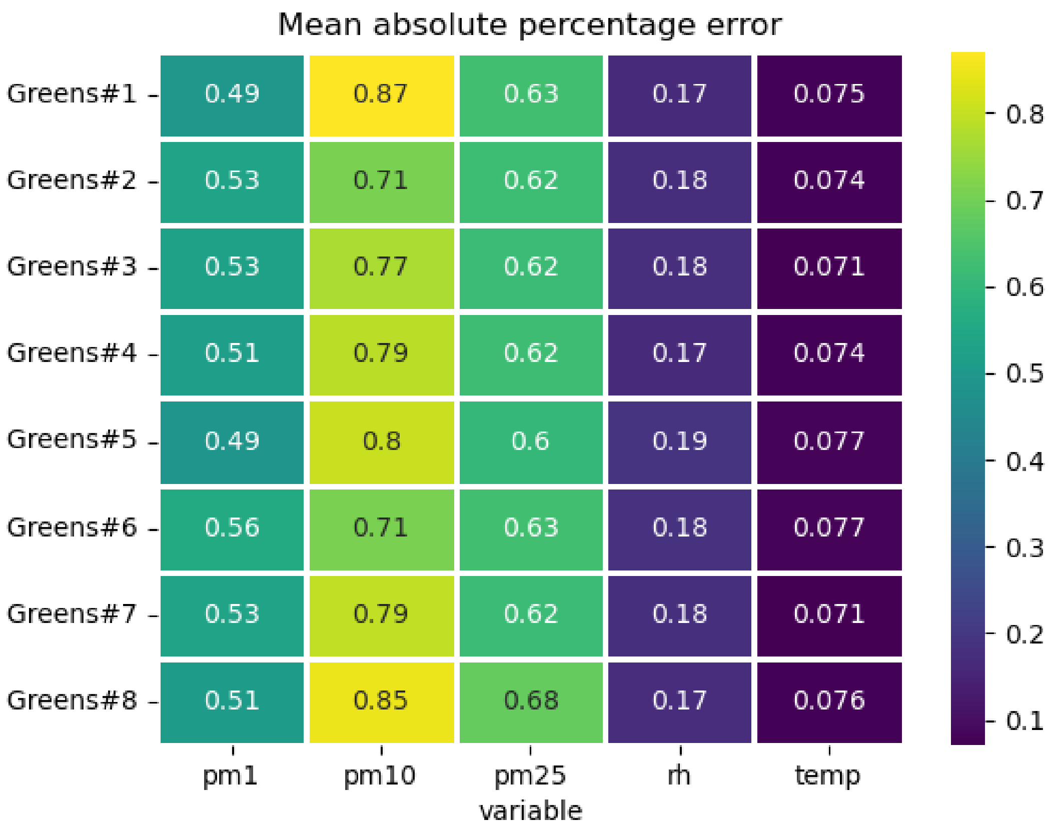

Figure S4: Heatmap of the mean absolute percentage error of the uncalibrated low-cost monitors by the measured variables.

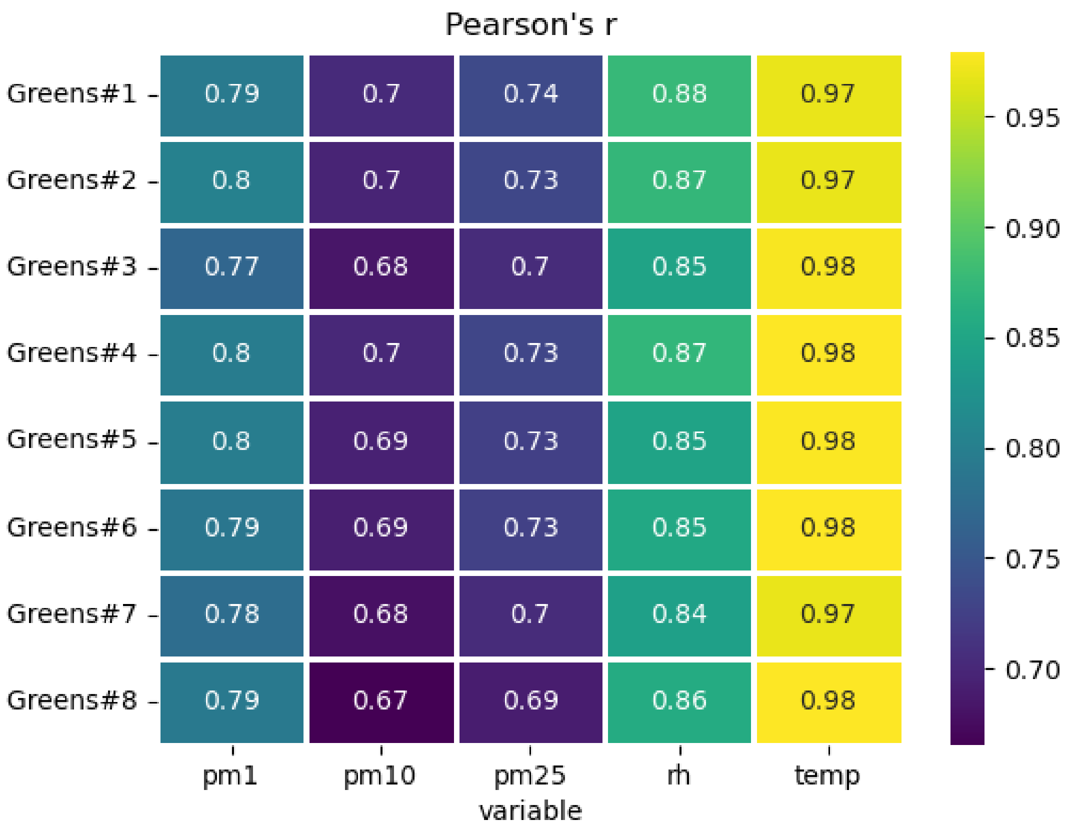

Figure S5: Heatmap of the Pearson's r of the uncalibrated low-cost monitors by the measured variables.

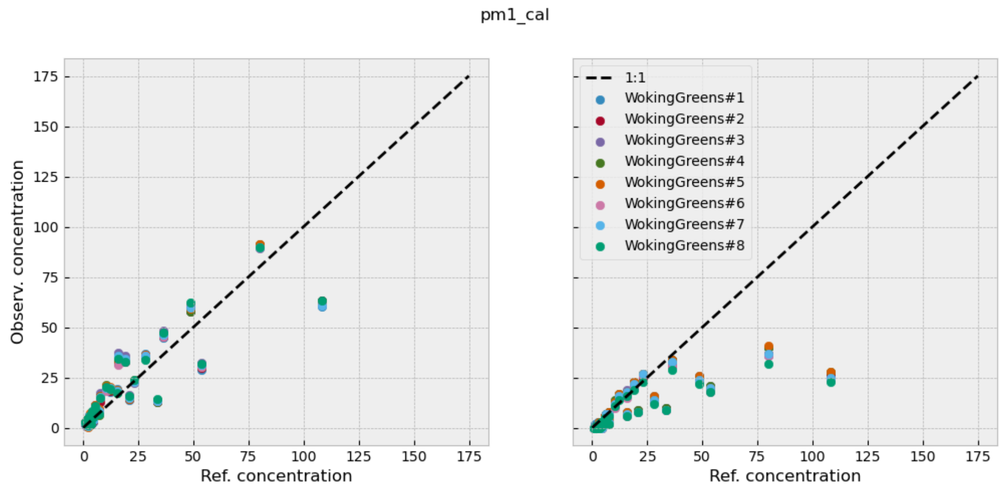

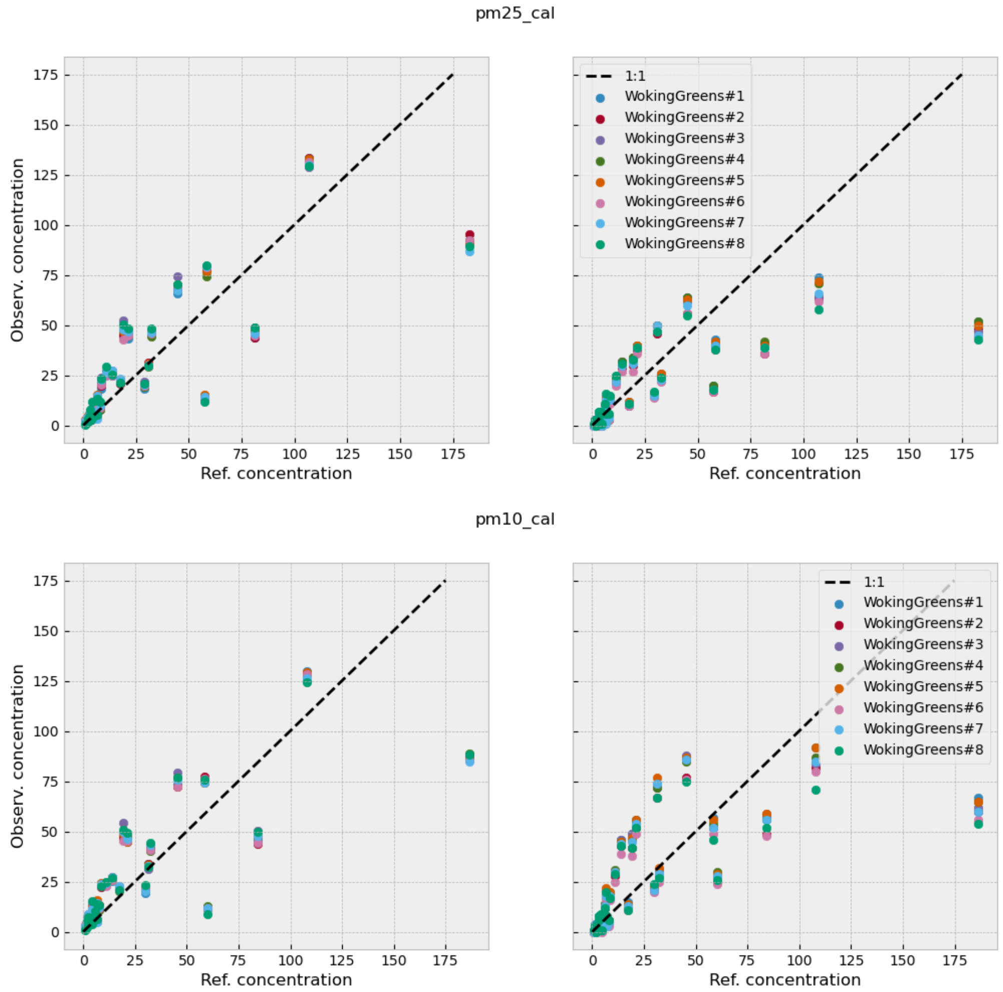

Figure S6: Calibration results for the monitors. The left graphs show the calibrated data and the right graphs show the original data.

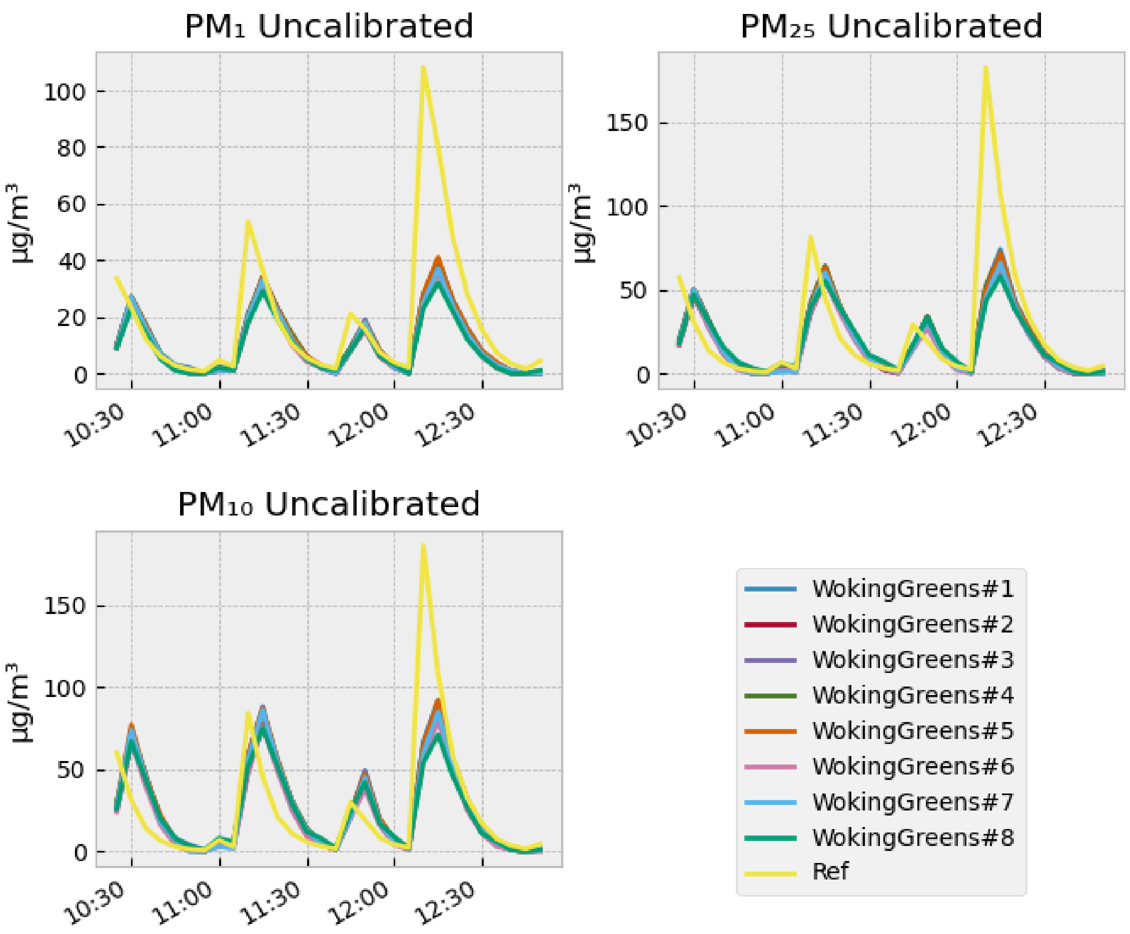

Figure S7: Data of the uncalibrated PM monitors in relation to the reference sensor.

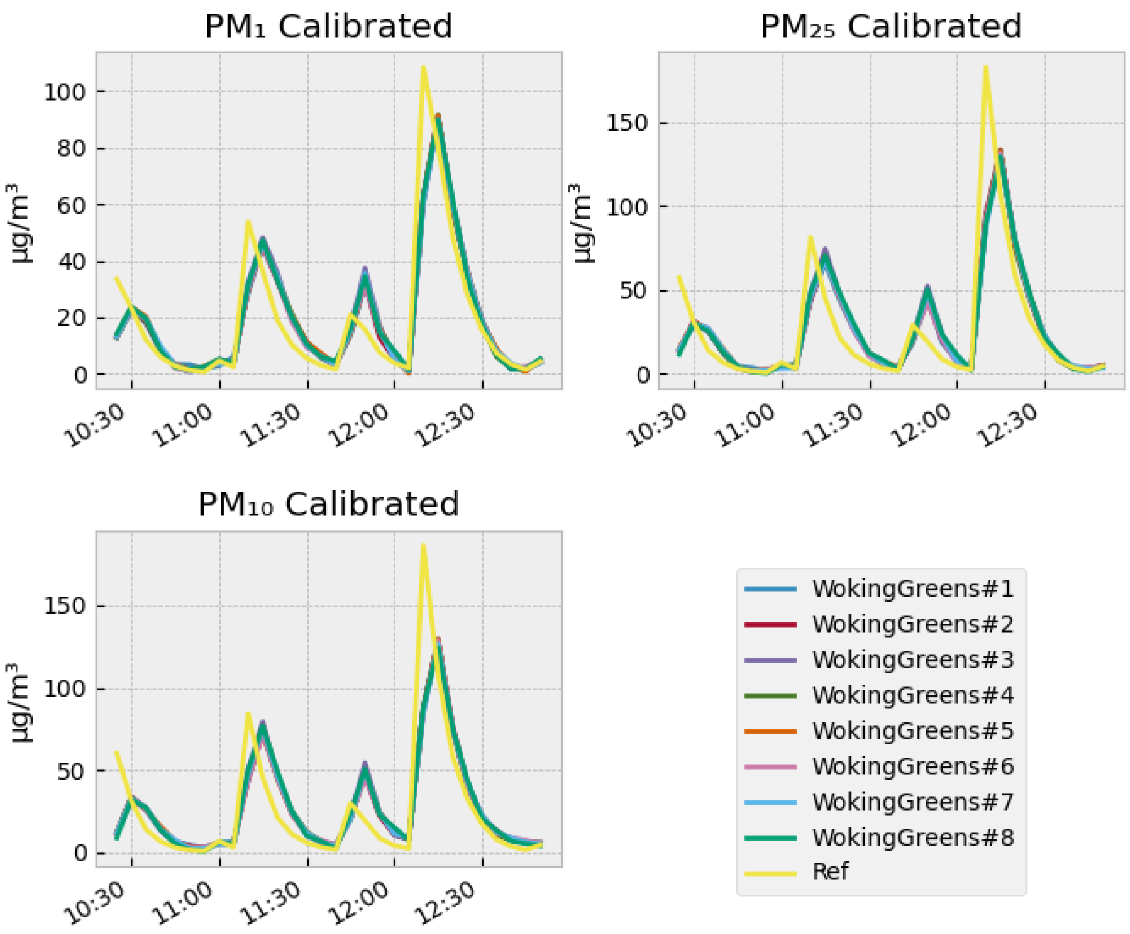

Figure S8: Data of the PM monitors in relation to the reference sensor after calibration.

Features description and sources

Table S2: Features description and sources.

Feature	Name in the model	Type	Description	Source
particulate matter concentration	pm1, pm25, pm10	target variable	LCM PM concentration	Davis® AirLink
mean target PM concentration	ymean	temporal	average of PM concentrations	Davis® AirLink
day of the week	day_of_week	temporal	day of the week from Monday to Sunday (0 to 7)	-
month	month	temporal	month, with January as 0	-

			and December as 11	
wind speed	u10, and v10, with a prefix indicating the direction of the data in relation to the city (e.g. sw_u10, w_v10, e_u10, nw_v10)	temporal	At 10 m from the ground, in m/s	ERA5
dewpoint temperature	d2m, with the same prefixes as wind speed	temporal	At 2 m from the ground, in Kelvin	ERA5
temperature	t2m, with the same prefixes as wind speed	temporal	At 2 m from the ground, in Kelvin	ERA5
boundary layer dissipation	bld, with the same prefixes as wind speed	temporal	Amount of kinect energy converted into heat by turbulence in the lower atmosphere, in J/m^2	ERA5
boundary layer height	blh, with the same prefixes as wind speed	temporal	In metres	ERA5
forecast surface roughness	fsr, with the same prefixes as wind speed	temporal	aerodynamic roughness length, in metres	ERA5
low cloud cover	lcs, with the same prefixes as wind speed	temporal	Fraction of cloud cover below approximately 2 km	ERA5
total cloud cover	tcc, with the same prefixes as wind speed	temporal	Fraction of cloud coverage	ERA5
total precipitation	tp, with the same prefixes as wind speed	temporal	Accumulated large-scale and convective precipitation, in metres	ERA5
surface net	ssr, with the	temporal	Difference of	ERA5

solar radiation	same prefixes as wind speed		incident solar radiation and the reflected by Earth’s surface	
mean total precipitation rate	mtpr, with the same prefixes as wind speed	temporal	In kg/m^2s	ERA5
wind angle	wind_angle	temporal	In degrees	Calculated from ERA5
local road density	A Road, B Road, Local Access Road, Local Road, Minor Road, Motorway, Restricted Local Access Road, and Secondary Access Road	spatial	Density of roads in a 200 m^2 square around each point. By road type.	OS
3 nearest monitor PM concentration	nearest_monitor_pm10, nearest_monitor_pm25, nearest_monitor_pm1, with a suffix indicating the distance rank (e.g. nearest_monitor_pm10_0, nearest_monitor_pm10_2)	spatiotemporal	In ug/m^3	Davis® AirLink
3 nearest monitor distance	nearest_monitor_distance, with a suffix indicating the distance rank	spatiotemporal	In degrees	Davis® AirLink
3 nearest monitor angle	nearest_monitor_angle, with a suffix indicating the distance rank	spatiotemporal	In degrees	Calculated from the Davis® AirLink data
3 nearest monitor angle in relation to the wind	nearest_monitor_angle_wind, with a suffix indicating the distance rank	spatiotemporal	From 0 (the monitor being downwind) to 1 (upwind)	Calculated from the Davis® AirLink and ERA5 data

3 nearest monitors PM concentration interpolated	nearest_monitor_pm1_idw, nearest_monitor_pm25_idw, nearest_monitor_pm10_idw	spatiotemporal	Interpolation by IDW with the weight of 2	Calculated from the Davis® AirLink data
difference between the interpolated concentrations and the mean concentration	pm_idwvar	spatiotemporal	-	Calculated from the Davis® AirLink data

PM data description

In this section we present details of the PM concentrations measured by the low-cost monitors. Figure S9 show a comparison between the monitors mean PM concentrations. The values are in respect to the average of all monitors. Figure S10 show the PM_{10} concentration distribution in the dataset of each monitor. Table S3 presents the quartile distribution of the monitors' data. Figure S11 show the calibrated PM concentration over time by monitor. Table S4 presents a statistical description of the PM data by monitor. Figure S12 shows a heatmap of the average difference between each monitor for each interquartile, where the rows and columns are respective to the monitor`s number.

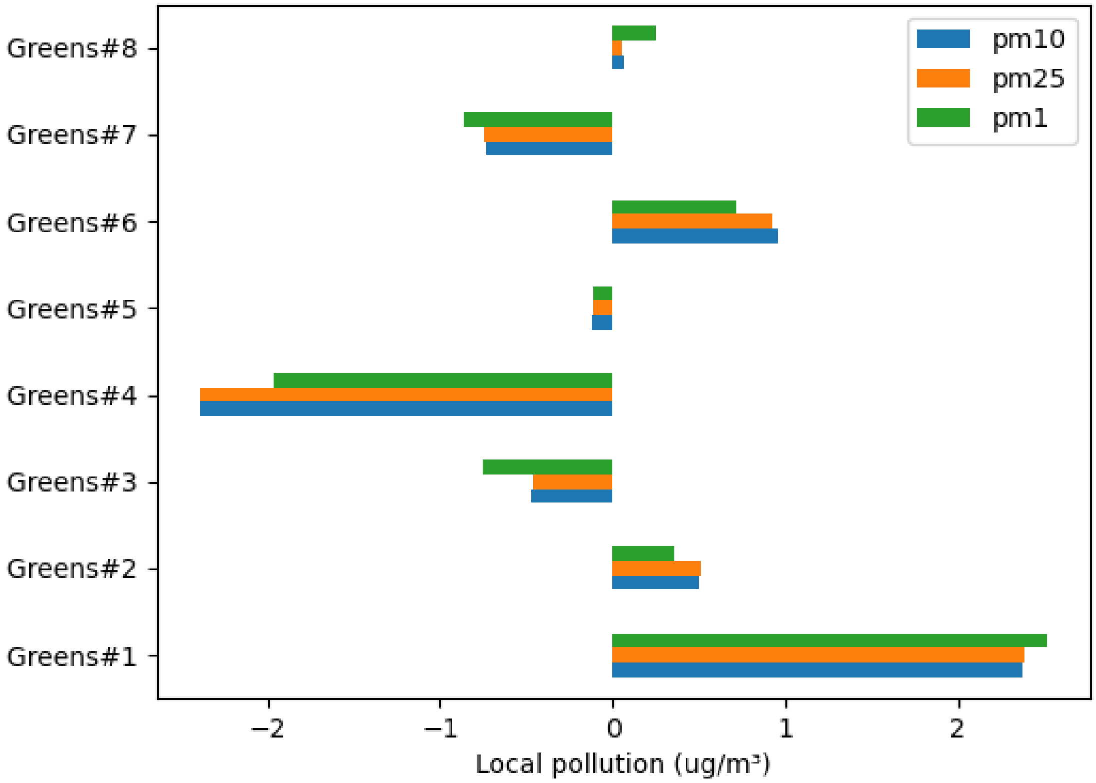

Figure S9: Comparison of the mean difference between the PM concentrations and the average between the monitors for each monitor.

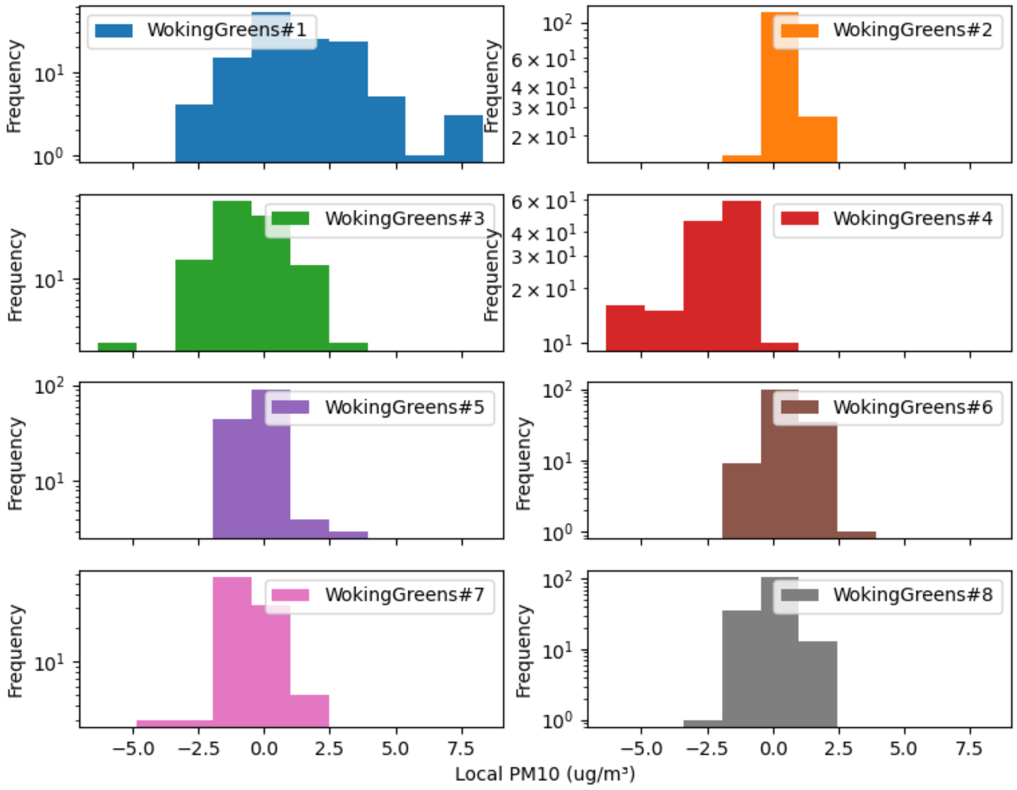

Figure S10: Histograms of the difference between the PM_{10} concentrations and the average between the monitors for each monitor.

Table S3: Minimun, first, secont, third quartile and maximun values of PM data.

Monitor	1	2	3	4	5	6	7	8
PM_1	0.66/5.62/8.47/17.43/46.82	1.64/4.69/7.71/14.00/37.18	0.27/3.64/6.78/13.29/35.18	1.11/3.71/5.79/11.24/26.97	1.93/4.70/7.43/13.76/35.00	1.80/4.86/8.13/14.48/39.79	1.09/3.22/5.45/9.92/32.61	2.17/5.20/7.96/13.08/40.61
$PM_{2.5}$	0.66/5.83/8.79/19.14/47.48	1.64/4.92/8.41/15.64/47.76	0.27/3.94/7.73/15.97/45.65	1.11/3.83/6.12/12.55/33.72	1.94/4.97/8.05/16.42/43.77	1.80/5.07/8.66/17.57/51.57	1.10/3.45/6.34/11.95/42.07	2.18/5.35/8.34/14.43/48.83
PM_{10}	0.66/5.86/8.79/19.15/47.51	1.65/4.93/8.42/15.69/47.95	0.27/3.94/7.74/15.98/45.82	1.13/3.83/6.12/12.61/33.89	1.96/4.98/8.08/16.49/43.99	1.84/5.13/8.75/17.65/51.89	1.13/3.48/6.35/12.01/42.35	2.23/5.41/8.39/14.51/49.15

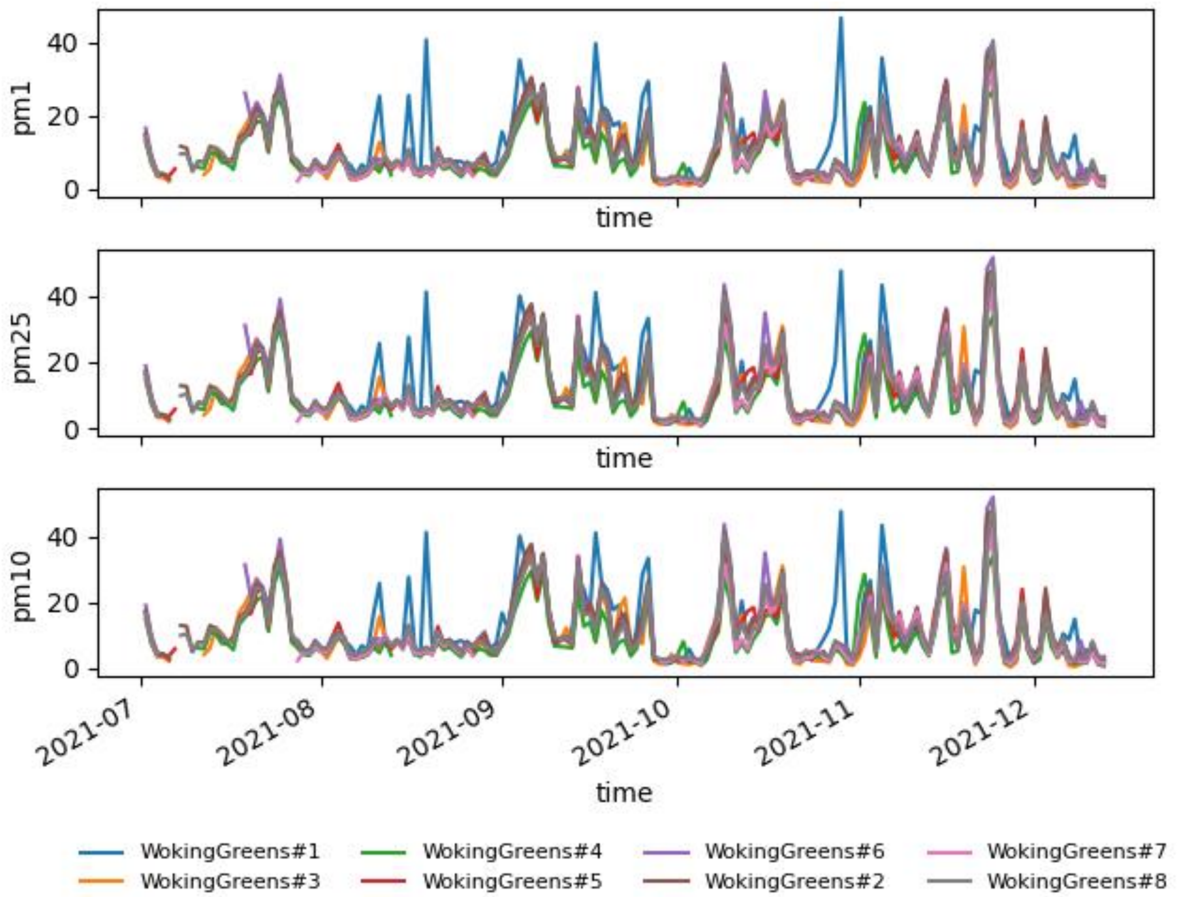

Figure S11: PM concentration over time, from the calibrated monitors data. The concentrations are in µg/m³. Each colour represents one monitor.

Table S4: Mean, median, and standard deviation of PM. The numbers are in a "mean / median (standard deviation)" format. The PM values are in µg/m³.

Monitor number	PM_1	$PM_{2.5}$	PM_{10}
1	12.29 / 8.47 (9.62)	13.55 / 8.79 (10.95)	13.58 / 8.79 (10.98)
2	10.29 / 7.71 (7.64)	11.81 / 8.42 (9.63)	11.84 / 8.42 (9.66)
3	9.22 / 6.79 (7.55)	10.91 / 7.73 (9.52)	10.93 / 7.74 (9.55)
4	8.11 / 5.79 (6.17)	9.10 / 6.12 (7.54)	9.14 / 6.12 (7.56)
5	9.93 / 7.43 (7.13)	11.36 / 8.05 (8.88)	11.39 / 8.08 (8.91)
6	10.73 / 8.13 (8.12)	12.36 / 8.66 (10.23)	12.43 / 8.75 (10.28)
7	7.57 / 5.45 (6.19)	8.83 / 6.34 (7.82)	8.87 / 6.35 (7.85)
8	10.26 / 7.96 (7.10)	11.45 / 8.34 (8.78)	11.50 / 8.39 (8.82)

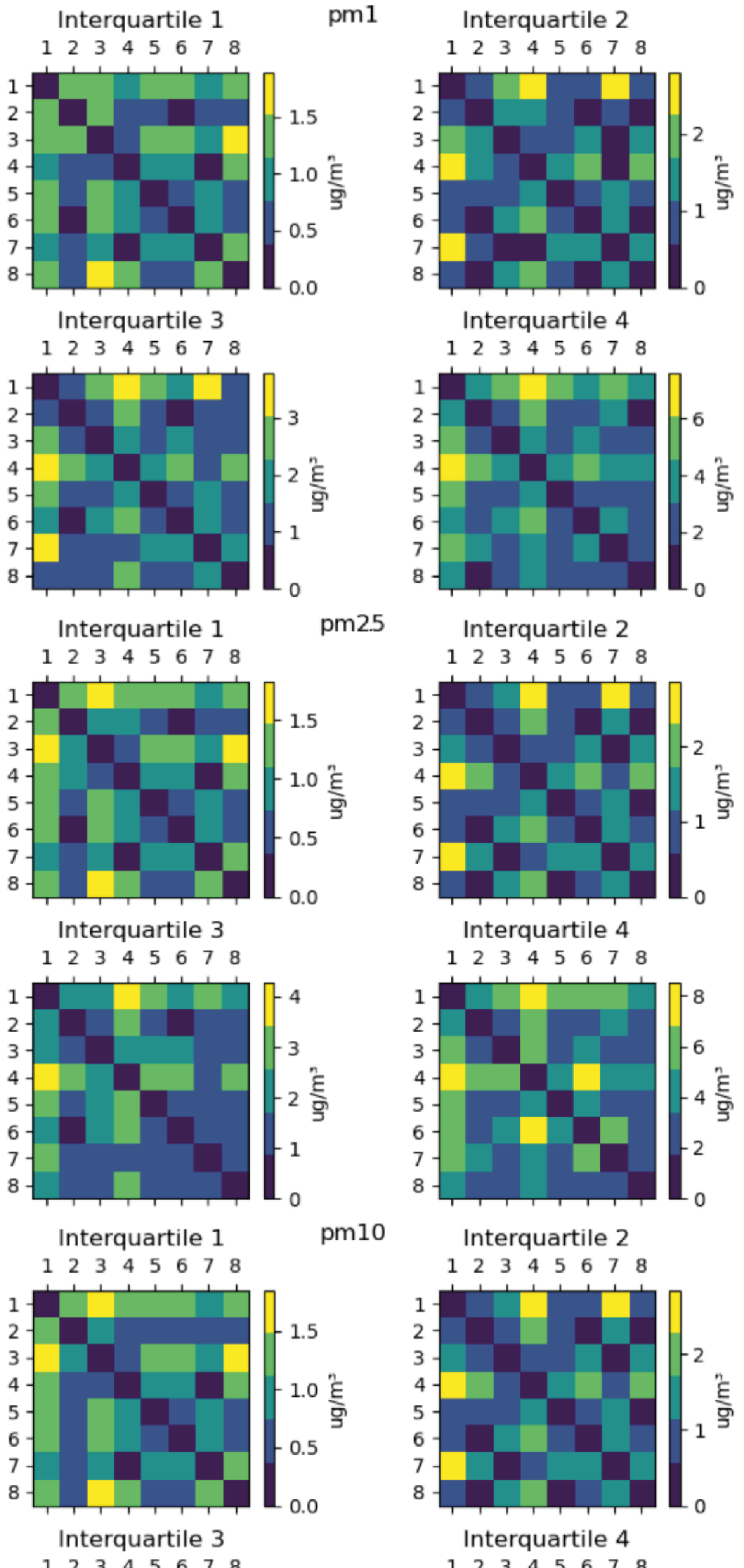
pm1
Interquartile 1
Interquartile 2
Interquartile 3
Interquartile 4
pm25
Interquartile 1
Interquartile 2
Interquartile 3
Interquartile 4
pm10
Interquartile 1
Interquartile 2
Interquartile 3
Interquartile 4
ug/m³

Figure S12: Average difference of PM_1, $PM_{2.5}$, and PM_{10} concentrations between the monitors for each interquartile of the data. The rows and columns are respective to the monitor`s number. The difference is expressed by the colour of the cell. The diagonal is zero because the difference between the monitor and itself is zero.

The ML models

In order to maintain a reasonable length in the manuscript, the figures of the evaluation of the PM2.5 and PM10 are presented here (Figures S11 and S12) alongside their sanity tests (Figures S13 and S14).

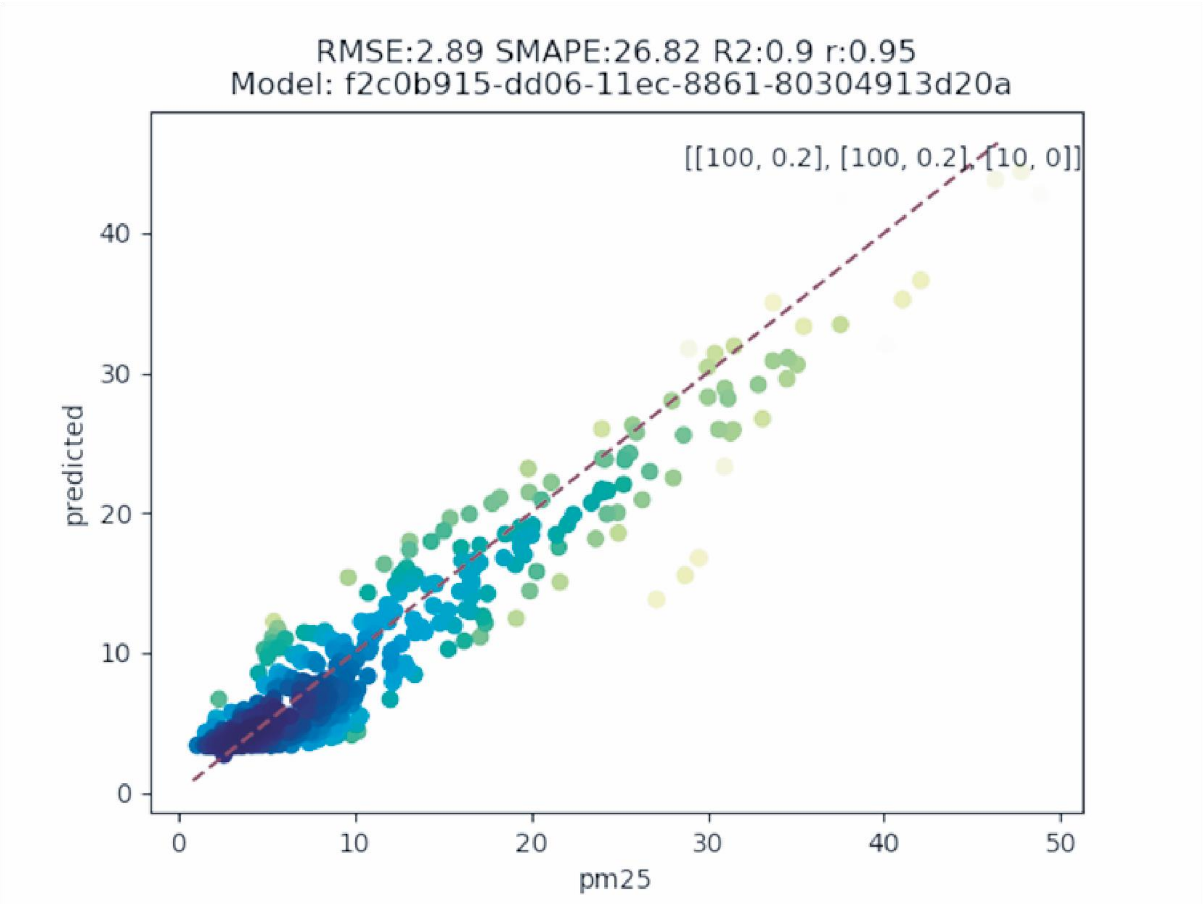

Figure S13: Comparison between the values of $PM_{2.5}$ predicted by the model (y axis) and the actual values (x axis) in the evaluation dataset. The color of the dots are porportional to the density of points. The dashed line is the 1:1 line. The number of neurons per layer and dropout rate are in the top-right corner in the format “[number of neurons, dropout rate]”. The evaluation metrics are displayed over the figure, and the name of the model is below it.

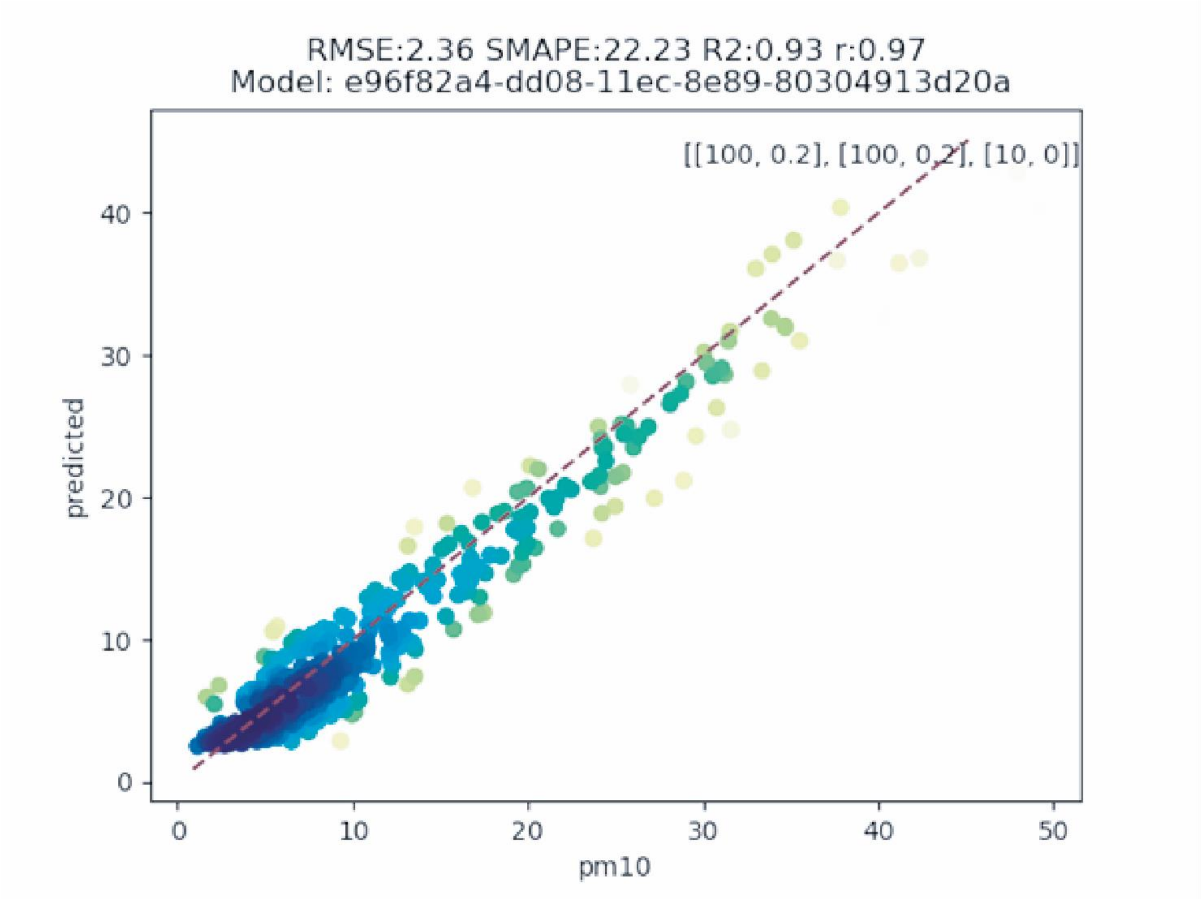

Figure S14: Comparison between the values of PM_{10} predicted by the model (y axis) and the actual values (x axis) in the evaluation dataset. The color of the dots are porportional to the density of points. The dashed line is the 1:1 line. The number of neurons per layer and dropout rate are in the top-right corner in the format "[number of neurons, dropout rate]". The evaluation metrics are displayed over the figure, and the name of the model is below it.

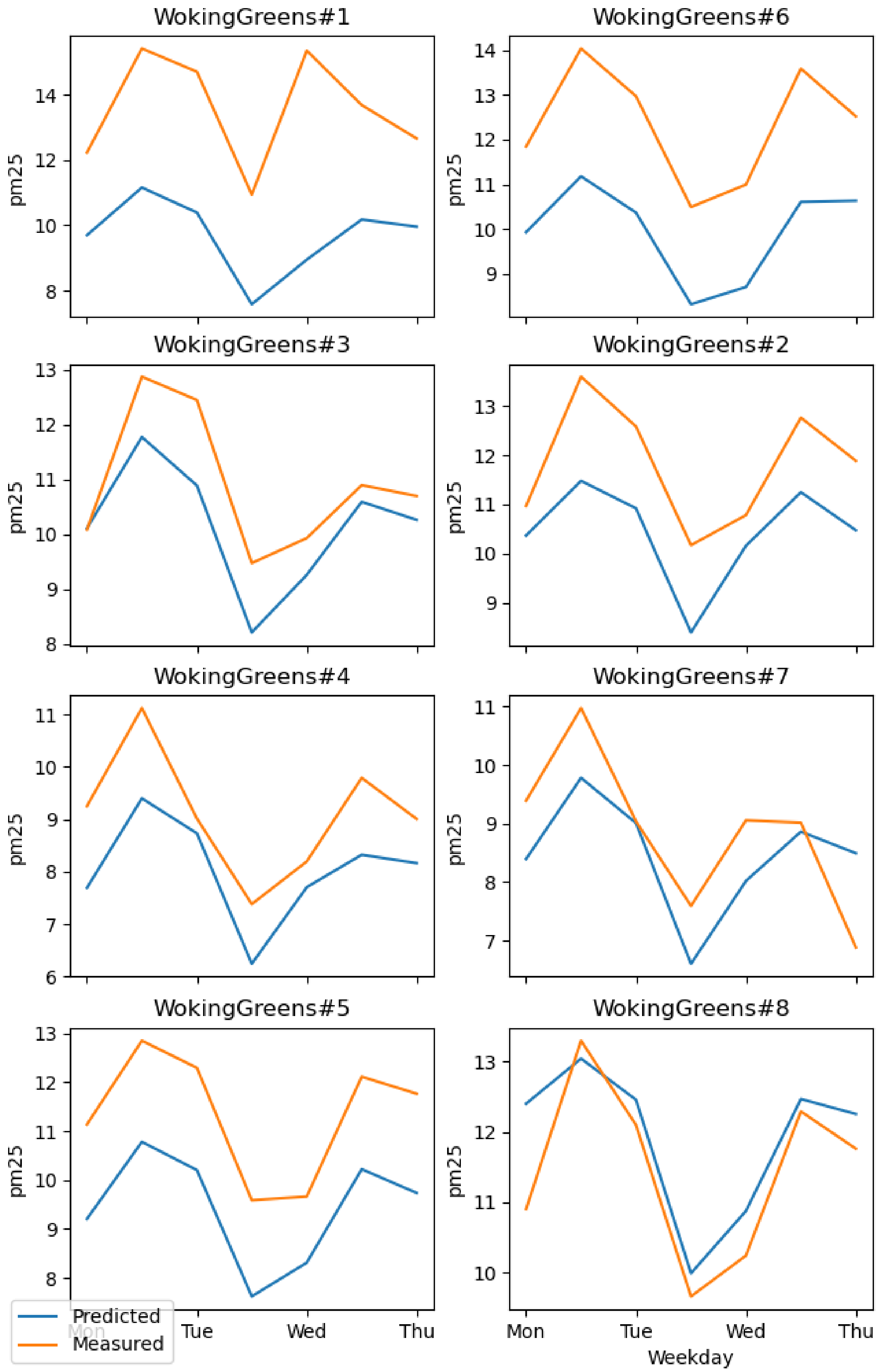

Figure S15: Comparison of the weekly variation of $PM_{2.5}$ between the model and the dataset. Stations numbers 2, 7 and 8 are the evaluation ones. The rest belongs to the training set.

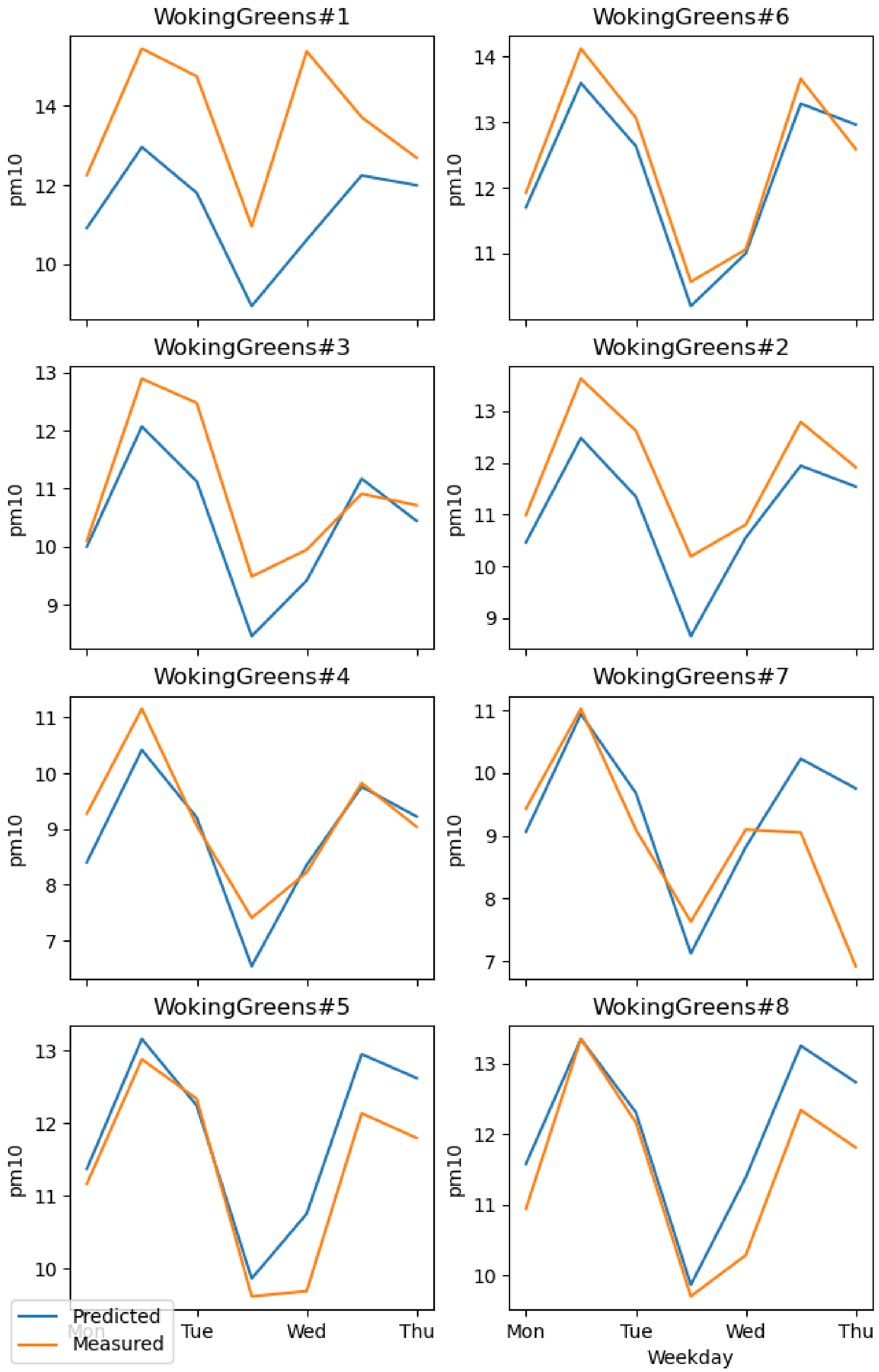

Figure S16: Comparison of the weekly variation of PM_{10} between the model and the dataset. Stations numbers 2, 7 and 8 are the evaluation ones. The rest belongs to the training set.

Sensitivity analysis

The algorithm used consists of the following steps:

1. Calculate the min, max and median values of the training set features.
2. Calculate the model output with the median values calculated in step 1.
3. Define a resolution constant (10 in this work).
4. Calculate an array of values equally spaced between the min and max values calculated in step 1, with a size equal to the constant defined in step 3.
5. For each feature, calculate the model output using the median values calculated in step 1, swapping the values of the target feature with each value of the array calculated in step 4.
6. For each feature, calculate the absolute difference between the output values calculated in step 5 and the output values calculated in step 2, and divide by the constant defined in step 3.
7. For each feature, sum the values calculated in step 6.

The values of the analysis cannot be directly applied in other studies, nor can be used to extrapolate physical meaning. It can be used as a tool to rank the features in order of importance considering only their linear influence on the model. The full analysis output is available in Figures S15, S16, and S17.

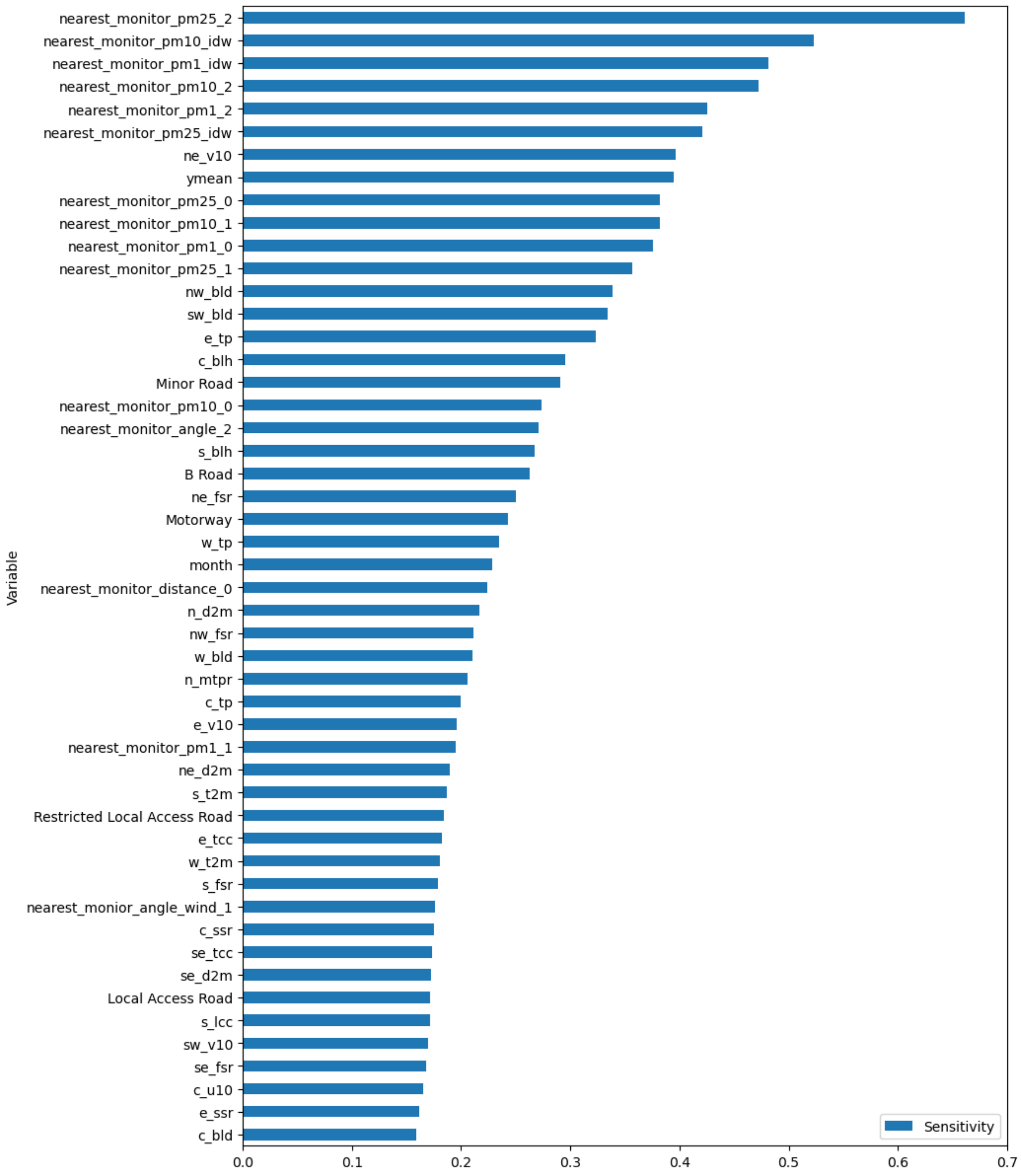

Figure S17: Sensitivity analysis of the PM_1 model. (Continue below)

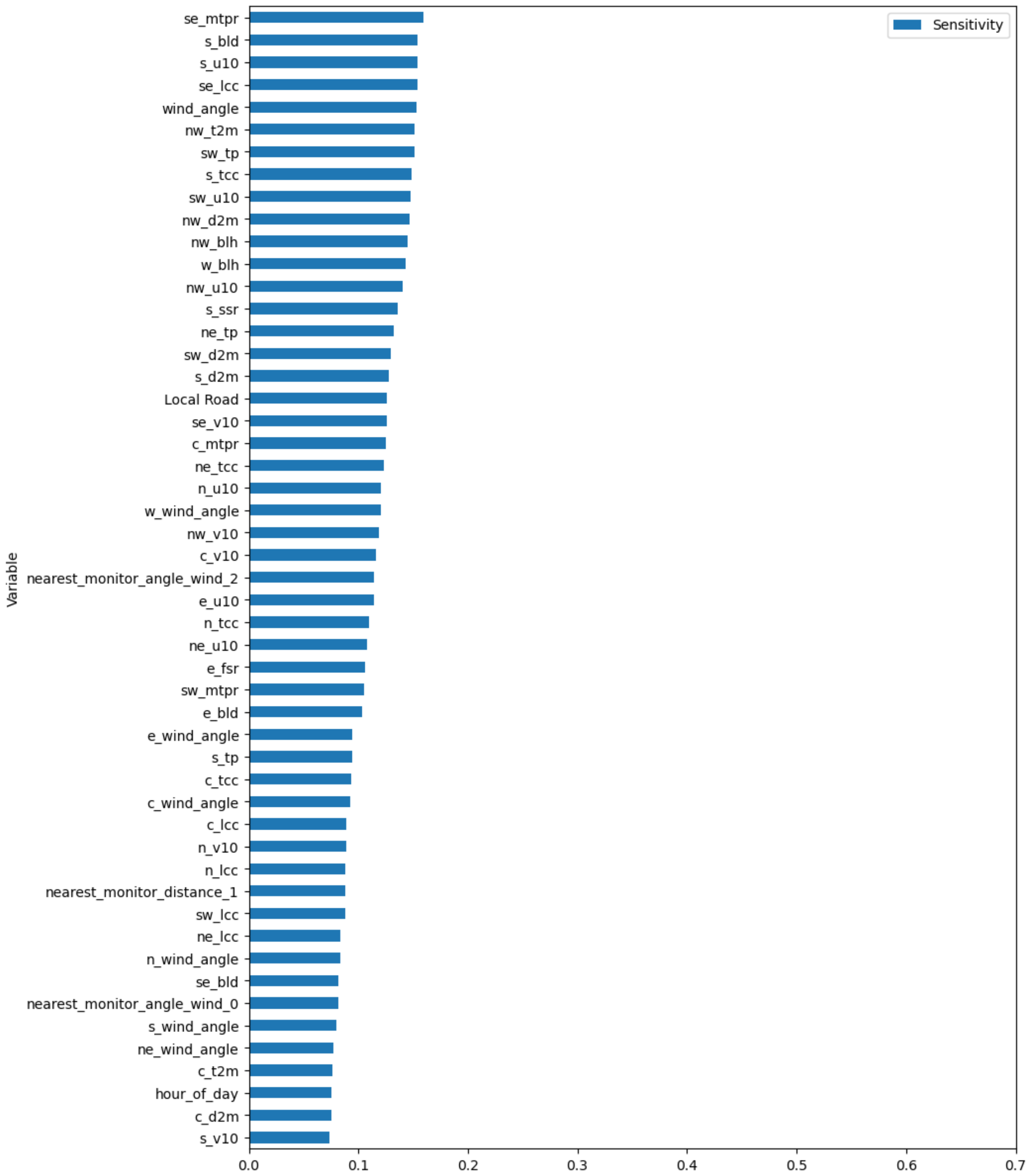

Figure S17: Sensitivity analysis of the PM_1 model. (Continue below)

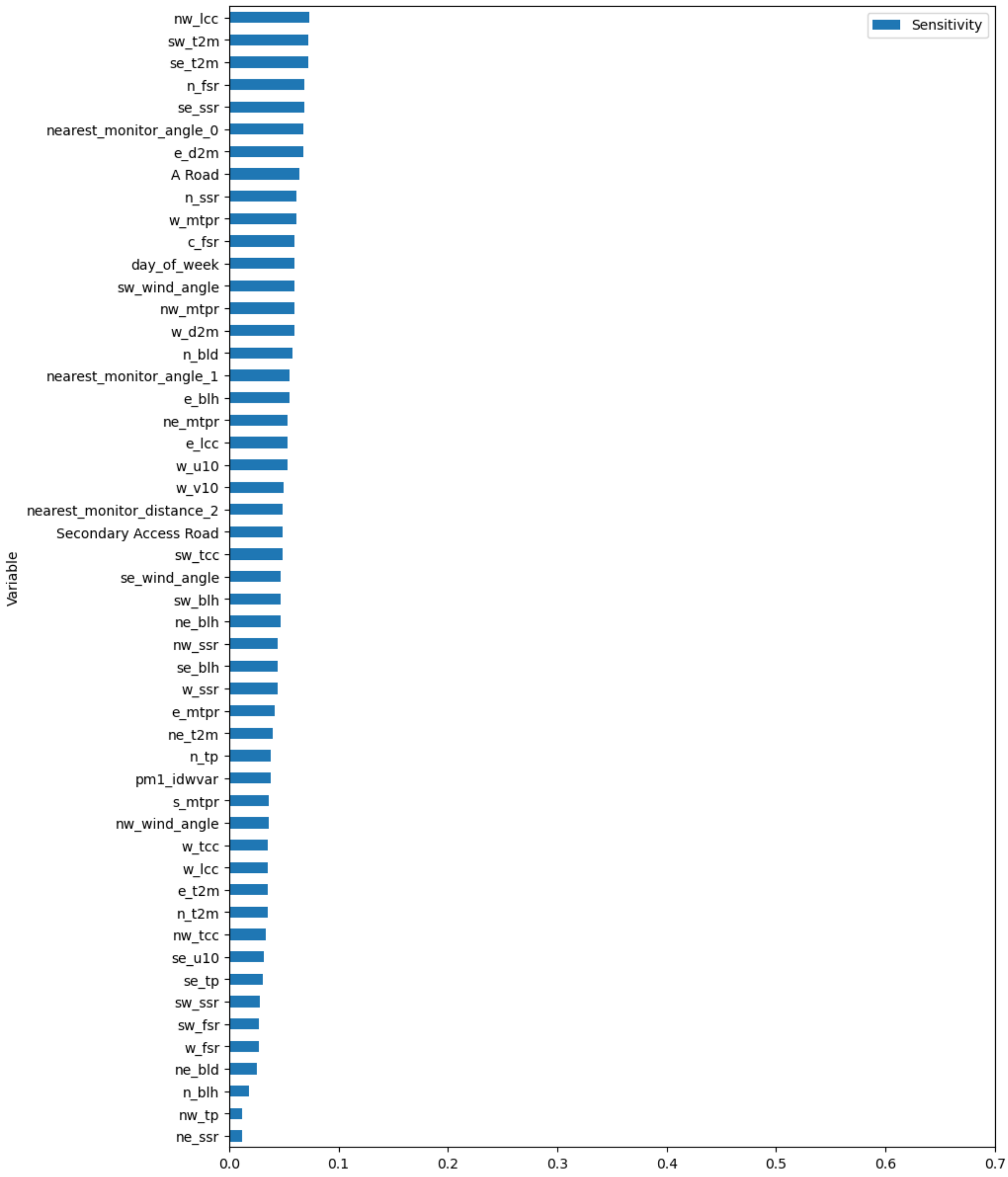

Figure S17: Sensitivity analysis of the PM_1 model.

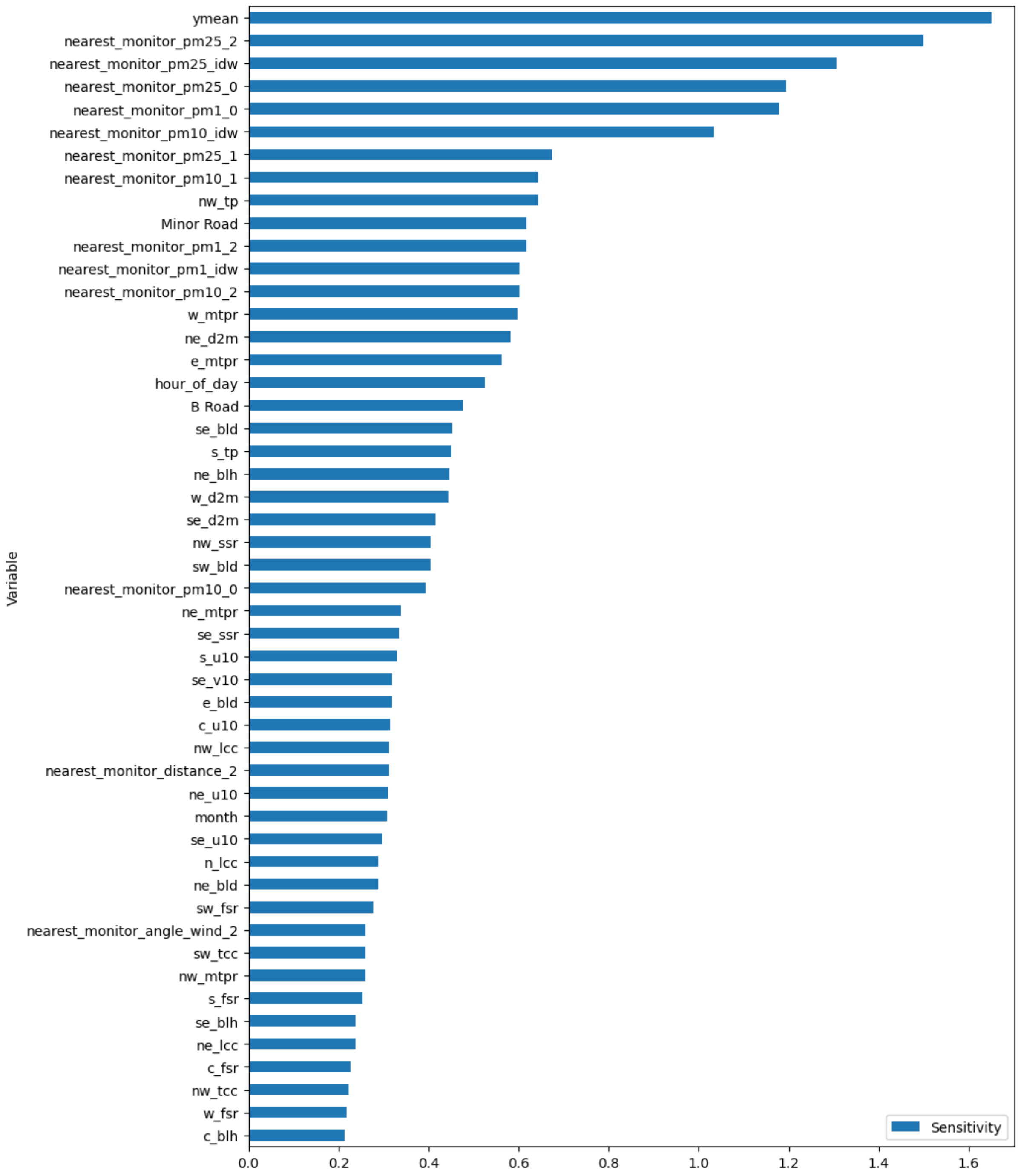

Figure S18: Sensitivity analysis of the $PM_{2.5}$ model. (Continue below)

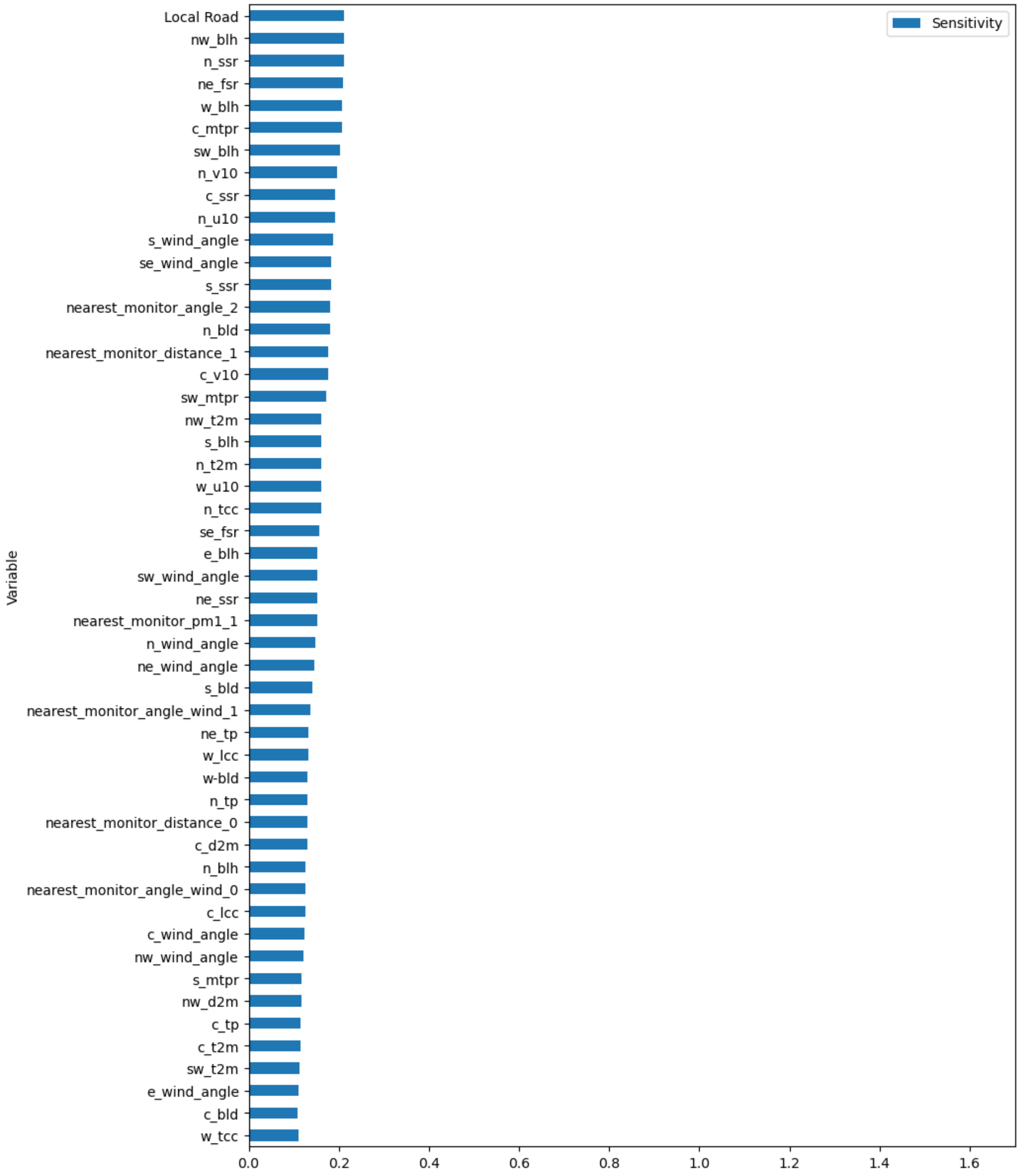

Figure S18: Sensitivity analysis of the $PM_{2.5}$ model. (Continue below)

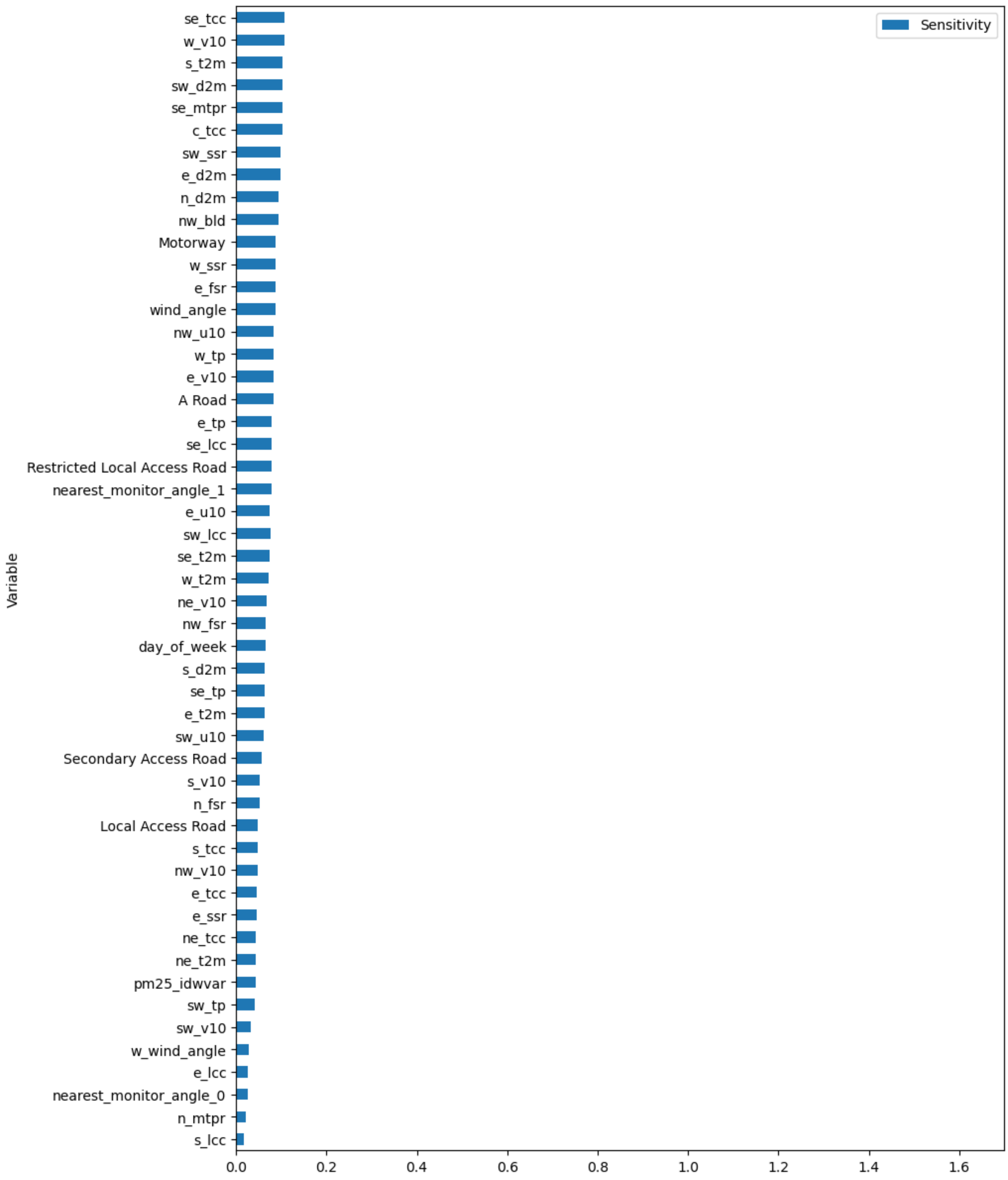

Figure S18: Sensitivity analysis of the $PM_{2.5}$ model.

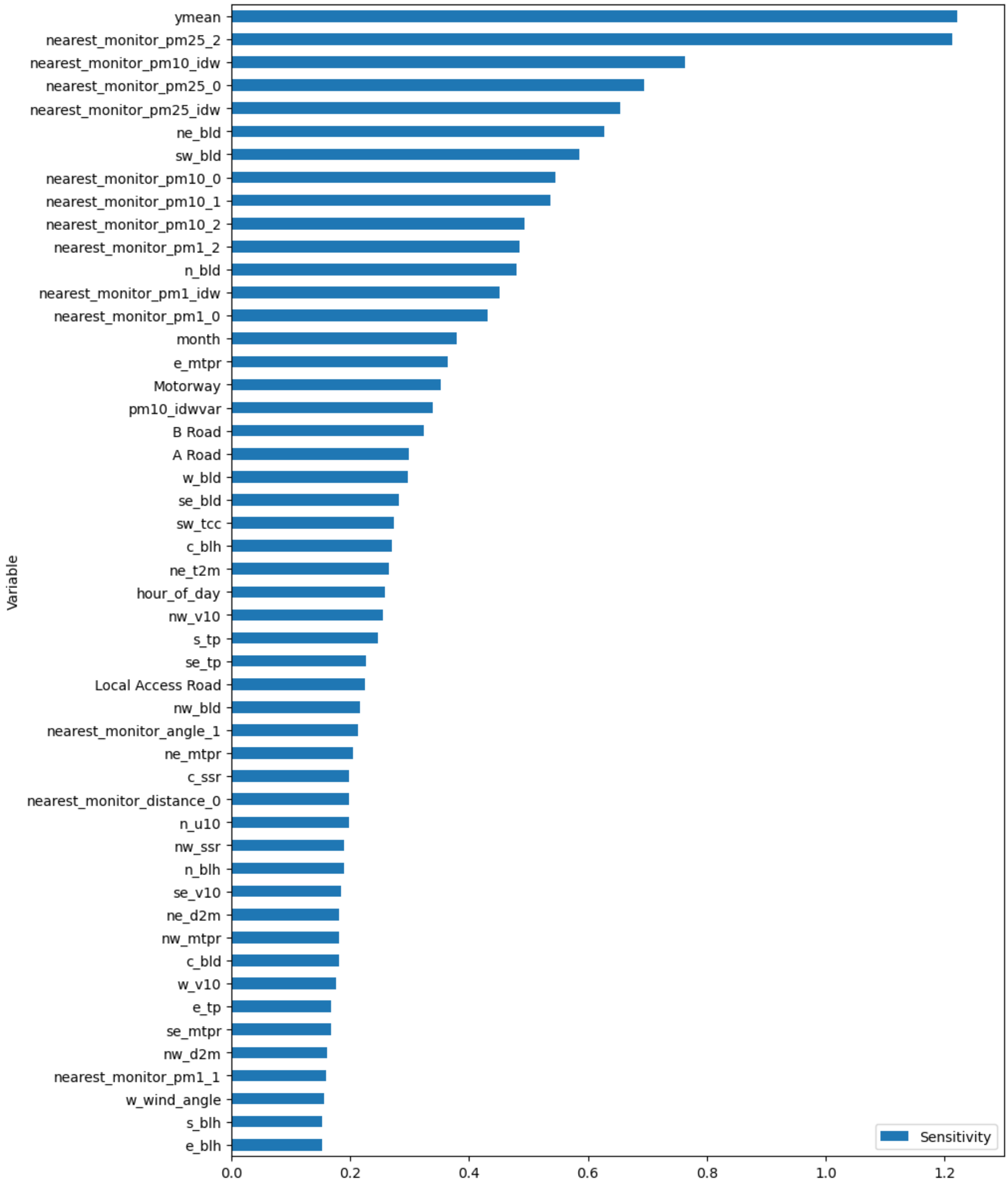

Figure S19: Sensitivity analysis of the PM_{10} model. (Continue below)

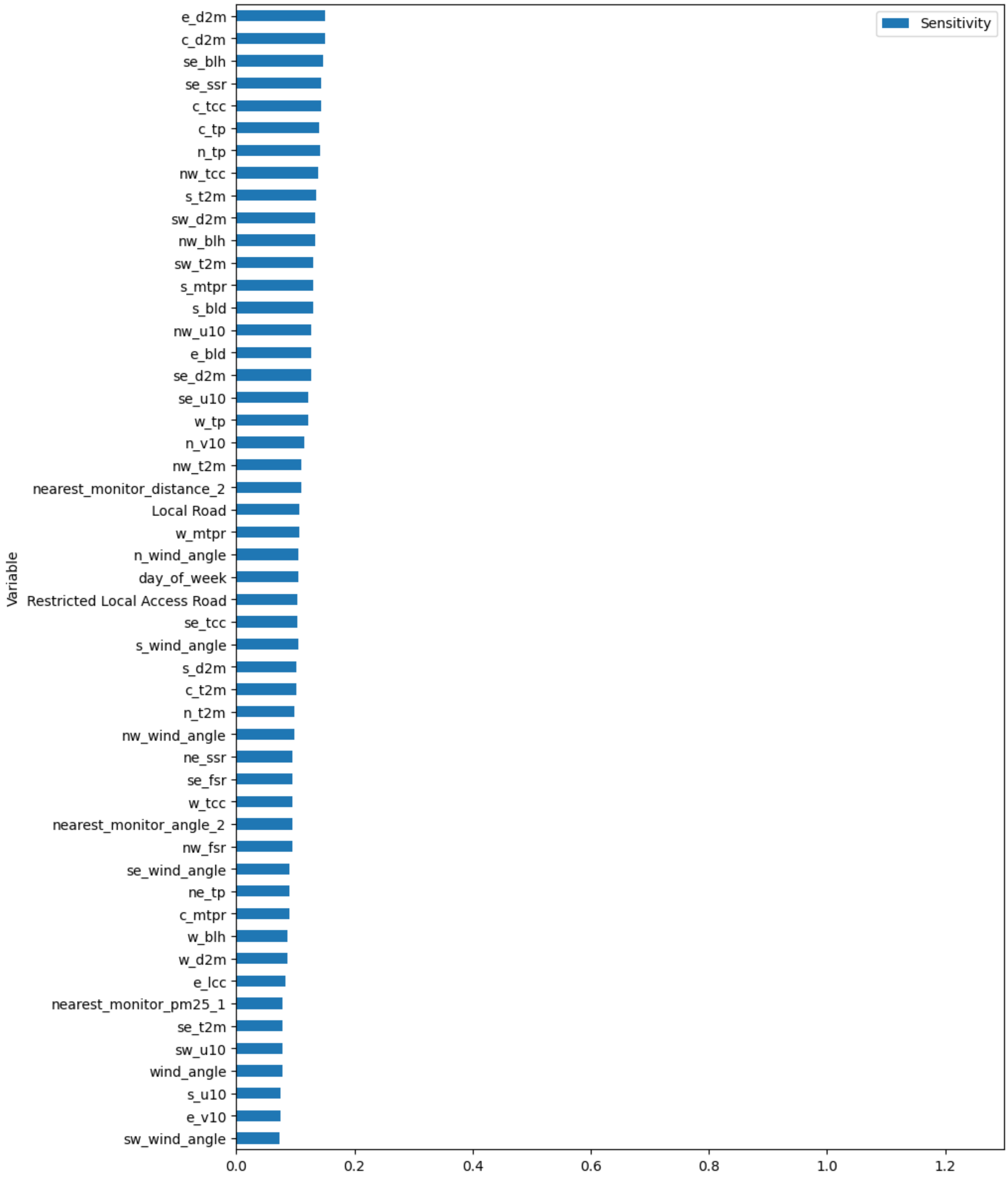

Figure S19: Sensitivity analysis of the PM_{10} model. (Continue below)

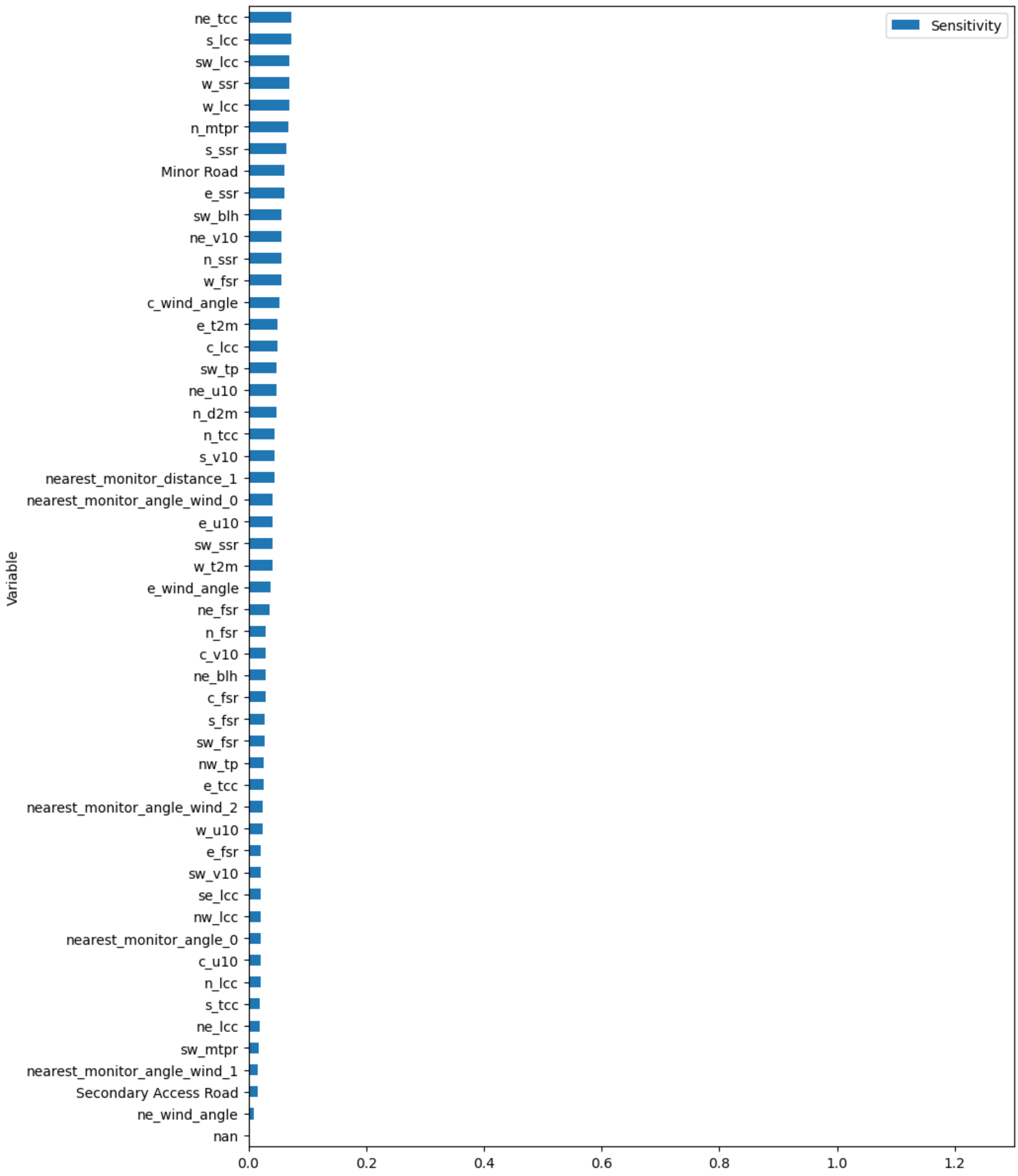

Figure S19: Sensitivity analysis of the PM_{10} model.

Early version of the PM_1 model

Figures illustrating the evalyation of the early version of the PM_1 model are presented here (Figures S18 and S19). The quantity of points in the evaluation dataset is higher due the use of one hour frequency. The frequency also allow the sanity test by the daily variation (Figure S19, left), where the sub estimation of PM_1 concentration in the nighttime is visible.

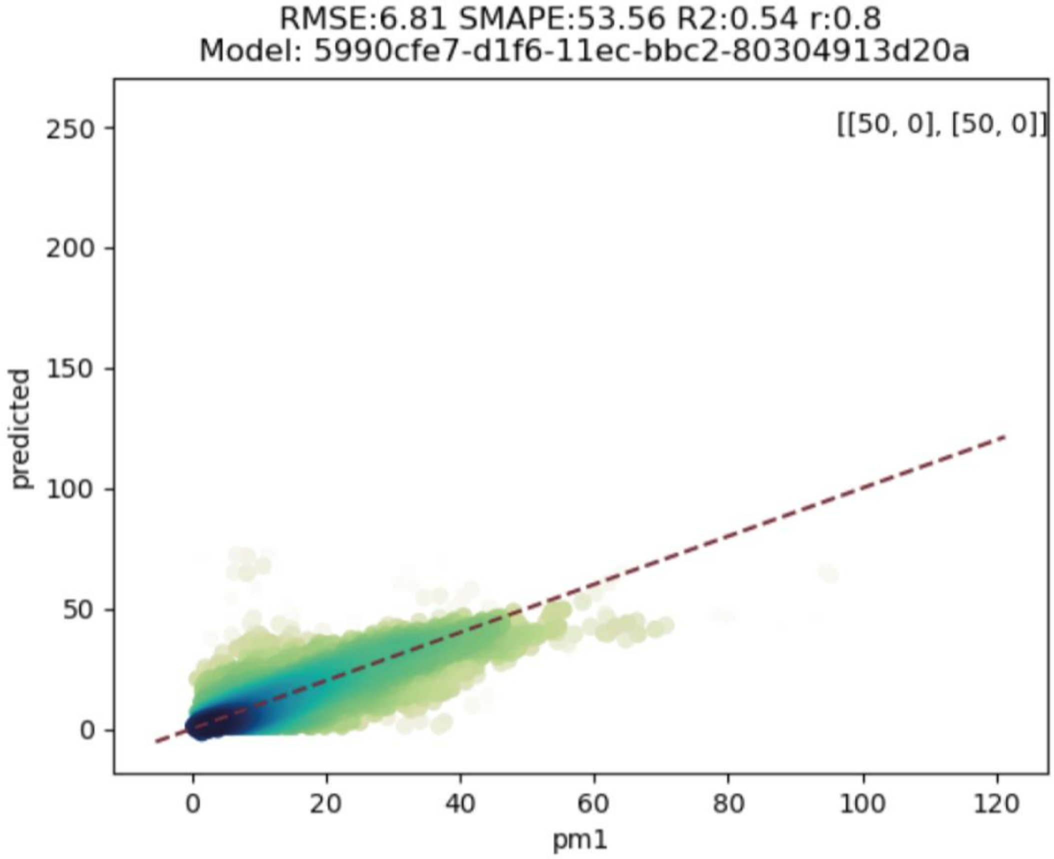

Figure S20: Comparison between the values of PM_1 predicted by the estou version of the model (y axis) and the actual values (x axis) in the evaluation dataset. The color of the dots are porportional to the density of points. The dashed line is the 1:1 line. The number of neurons per layer and dropout rate are in the top-right corner in the format "[number of neurons, dropout rate]". The evaluation metrics are displayed over the figure, and the name of the model is below it.

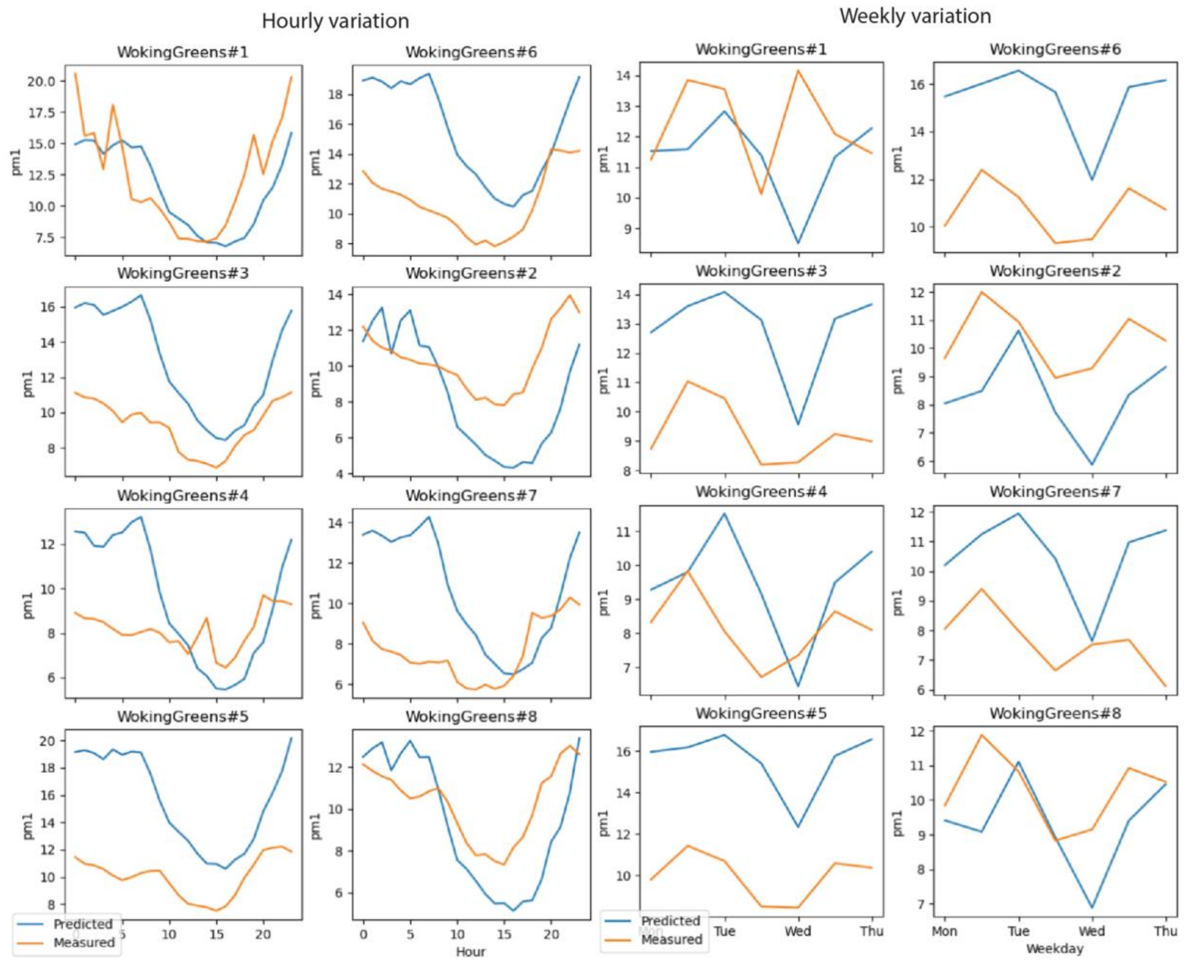

Figure S21: Comparison of the daily (left) and weekly (right) variation of PM_1 between the early version of the model and the dataset. Stations numbers 2, 7 and 8 are the evaluation ones. The rest belongs to the training set. The hour is in local time.

References

Chollet, F. (2015). keras.

Gillies, S., Taves, M., Arnott, J., Tonnhofer, O., Bossche, J. V., Wasserman, J., … & Hards, B. (2021). Toblerity/Shapely: Shapely 1.8.0 (1.8.0). Zenodo. https://doi.org/10.5281/zenodo.5597139

Harris, C. R., Millman, K. J., Van Der Walt, S. J., Gommers, R., Virtanen, P., Cournapeau, D., ... & Oliphant, T. E. (2020). Array programming with NumPy. *Nature*, *585*(7825), 357-362.

Hoyer, S., & Hamman, J. (2017). xarray: ND labeled arrays and datasets in Python. *Journal of Open Research Software*, *5*(1).

Hoyer, S., Roos, M., Hamman, J., keewis, Cherian, D., Fitzgerald, C., … & Wolfram, P. J. (2021). pydata/xarray: v0.20.1 (v0.20.1). Zenodo. https://doi.org/10.5281/zenodo.5648431

Hunter, J. D. (2007). Matplotlib: A 2D graphics environment. *Computing in science & engineering*, *9*(03), 90-95.

Jordahl, K., Bossche, J. V., Fleischmann, M., McBride, J., Wasserman, J., Badaracco, A. G., … & Wasser, L. (2021). geopandas/geopandas: v0.10.2 (v0.10.2). Zenodo. https://doi.org/10.5281/zenodo.5573592

McKinney, W. (2010, June). Data structures for statistical computing in python. In Proceedings of the 9th Python in Science Conference (Vol. 445, No. 1, pp. 51-56).

Omidvarborna, H., Kumar, P., & Tiwari, A. (2020). 'Envilution™'chamber for performance evaluation of low-cost sensors. *Atmospheric Environment*, *223*, 117264.

Pedregosa, F., Varoquaux, G., Gramfort, A., Michel, V., Thirion, B., Grisel, O., ... & Duchesnay, E. (2011). Scikit-learn: Machine learning in Python. *the Journal of machine Learning research*, *12*, 2825-2830.

Reback, L., jbrockmendel, McKinney, W., Bossche, J. V., Augspurger, T., Cloud, P., … & Seabold, S. (2022). pandas-dev/pandas: Pandas 1.4.1 (v1.4.1). Zenodo. https://doi.org/10.5281/zenodo.6053272
